# HDAC10 and its implications in Sézary syndrome pathogenesis

**DOI:** 10.3389/fcell.2025.1480192

**Published:** 2025-01-31

**Authors:** Monika Pieniawska, Karolina Rassek, Bogumiła Skwara, Magdalena Żurawek, Iwona Ziółkowska-Suchanek, Lydia Visser, Monique Lodewijk, Małgorzata Sokołowska-Wojdyło, Berenika Olszewska, Roman J. Nowicki, Tomasz Stein, Aleksandra Dańczak-Pazdrowska, Adriana Polańska, Marta Szymoniak-Lipska, Natalia Rozwadowska, Katarzyna Iżykowska

**Affiliations:** ^1^ Institute of Human Genetics, Polish Academy of Sciences, Poznań, Poland; ^2^ Department of Pathology and Medical Biology, University of Groningen, University Medical Center Groningen, Groningen, Netherlands; ^3^ Department of Dermatology, Venereology and Allergology, Faculty of Medicine, Medical University of Gdańsk, Gdańsk, Poland; ^4^ Department of Dermatology, Venereology and Allergology, University Clinical Centre, Gdańsk, Poland; ^5^ Department of Dermatology, Poznań University of Medical Sciences, Poznań, Poland; ^6^ Department of Dermatology and Venereology, Poznań University of Medical Sciences, Poznań, Poland

**Keywords:** HDAC10, Sézary syndrome, CTCL, histone deacetylases, epigenetic regulators

## Abstract

Cutaneous T-cell lymphomas (CTCL) are a group of rare hematological malignancies characterized by infiltration of malignant T-cells into the skin. Two main types of CTCL constitute of Mycosis Fungoides (MF), a more indolent form of the disease, and Sézary syndrome (SS), the aggressive and leukemic variant with blood involvement. Sézary syndrome presents a significant clinical challenge due to its very aggressive nature, poor prognosis, and treatment resistance, and to date, the disease remains incurable. Histone deacetylase inhibitors have gained attention in CTCL treatment with promising results, but they expose limited specificity and strong side effects. Recent genomic studies underscore the role of epigenetic modifiers in CTCL pathogenesis, prompting an investigation into HDAC10, a member of class IIb HDACs, in SS. HDAC10 was investigated in different cancers, revealing its involvement in cell cycle regulation, apoptosis, and autophagy, but its role in CTCL is unknown. In this study we aimed to determine the role of HDAC10 in SS, focusing on its cellular localization, role in cell growth, and therapeutic potential. We indicated that HDAC10 is overexpressed in SS patients and located mainly in the cytoplasm. Its overexpression leads to an inhibitory effect on apoptosis progression when exposed to the pro-apoptotic compound Camptothecin (CPT). Knockdown of HDAC10 resulted in reduced cell growth and induction of apoptosis and autophagy, highlighting its potential importance in CTCL pathogenesis. Whole transcriptome analysis indicated that HDAC10 is associated with crucial cancer-related pathways, for example, hematopoietic cell lineage, PI3K-Akt signaling pathway, Ras signaling pathway, MAPK signaling pathway or JAK-STAT signaling pathway, which are critical for the survival and proliferation of malignant T cells. Inhibition of HDAC10 with selective HDAC10i increased the sensitivity of Sézary cells to the pro-apoptotic CPT. Our findings demonstrate that HDAC10 plays a key role in the molecular background of Sézary syndrome, highlighting its importance in the cellular mechanisms of the disease.

## 1 Introduction

Cutaneous T-cell lymphomas (CTCL) are a group of non-Hodgkin’s lymphomas characterized by infiltration of malignant T-cells into the skin (Wozniak et al., 2010). The two main subtypes of the disease are Mycosis Fungoides (MF) and Sézary syndrome (SS) (Jawed et al., 2014a). MF is more indolent in its course, while SS is an aggressive and leukemic variant, characterized by erythroderma, lymphadenopathy, and the infiltration of malignant T lymphocytes (Sézary cells) in the skin and blood (Pulitzer, 2017; Yamashita et al., 2012). The incidence rate of CTCL is around 0.77 out of 100,000 and the 5-year survival of SS is estimated at around 30% (Rassek et al., 2023a). SS manifests as a clinical challenge due to its chronic course, poor prognosis, and treatment resistance. The disease mainly affects adults, and the age of diagnosis is 50–60 (Giordano and Pagano, 2022). To date, SS is considered incurable, and therapeutic strategies focus on relieving symptoms and slowing the progression of the disease (Jawed et al., 2014b).

In the last decades, the role of epigenetic modifications in the pathogenesis of CTCL was indicated - with an impact on histone deacetylases (HDACs) in the molecular background of the disease. Moreover, histone deacetylase inhibitors (HDACi) have gained interest as promising therapeutic targets for CTCL (Mann et al., 2007; Campas-Moya, 2009). HDACs are a group of proteins important for the regulation of gene expression, which is correlated with histone modifications (Pieniawska and Iżykowska, 2022). HDACs remove acetyl groups from histones, which in turn leads to chromatin condensation and transcriptional repression (Pieniawska and Iżykowska, 2022). So far, 18 HDACs have been identified in humans and divided into four groups: class I (HDAC1, 2, 3, 8), which are ubiquitously expressed; class IIa (HDAC4, 5, 7, 9); class IIb (HDAC6, 10); class III (sirtuins 1–7); and class IV (HDAC11), which show more tissue-specific expression patterns (New et al., 2012). HDAC inhibitors (HDACi) prevent transcriptional repression and are used in the treatment of cancer, including CTCL. One of the well-determined anti-cancer effects of HDACi is the induction of apoptosis as HDACi activates many apoptotic mechanisms and can cause DNA damage accumulation, reactive oxygen species (ROS) production, and downregulation of oncoproteins (Li and Seto, 2016; Li et al., 2020; Jiang et al., 2021). Additionally, HDACi are known to promote cell cycle arrest, inhibit proliferation, and influence autophagy in cancer, although their precise role in promoting or inhibiting this process remains unclear (Milazzo et al., 2020; Gammoh et al., 2012).

Despite global progress in the field of targeted therapies, the effectiveness of treatment for patients with CTCL is very low. The response rate to HDACi available in clinical practice ranges from 20% to 40% (Prince and Dickinson, 2012; Zhang and Zhang, 2021). The HDACi used commonly in the clinic are mainly pan-HDAC inhibitors, which are not target-specific and affect multiple HDACs - predominantly those in class I (e.g., Vorinostat targets HDACs 1, 2, 3, 6, 7, 11; Romidepsin targets HDACs 1, 2, 4, 6) (Clarke et al., 2019). This causes noticed clinical implications, including side effects ranging from nausea, vomiting, and fatigue to potentially life-threatening symptoms (Suraweera et al., 2018; Subramanian et al., 2010).

In addition to the existing literature and research on histone deacetylase inhibitors in CTCL, the whole-genome and transcriptome next-generation sequencing (NGS) confirmed the significant role of epigenetic modifiers in the pathogenesis of CTCL (Iżykowska et al., 2017). In this study we focused on one particular epigenetic regulator, HDAC10, and its role in SS. HDAC10 belongs to class IIb (Ridinger et al., 2018) and is involved in a variety of cellular processes, including cell cycle, apoptosis, autophagy, and is gaining attention for its potential role in cancer cell biology (Oehme et al., 2013; Li et al., 2015; Yang et al., 2016; Cheng et al., 2021). Moreover, presence of HDAC10 was indicated in many human tissues, including the liver, spleen, heart, testis, pancreas, brain, and placenta (Cheng et al., 2021). HDAC10 expression was also described in malignancies, for example, non-small cell lung carcinoma (NSCLC) and melanoma (Liu et al., 2020; Ling et al., 2024; Ling et al., 2023). Despite growing interest in HDAC10 in oncology, its role in CTCL is unknown and its involvement in the pathogenesis or treatment has not been investigated. The purpose of this study was to investigate the function of HDAC10 in SS, focusing on its localization, involvement in cell growth, and role as a potential therapeutic target.

## 2 Materials and methods

### 2.1 Cell lines

Two lymphoid cell lines were included in the study: Hut-78 (ATCC TIB-161) – established from a 53 year-old SS patient, and SeAx–established from the peripheral blood of a patient with SS - kindly provided by Markus Möbs (Kaltoft et al., 1987). Cell lines were cultured in RPMI1640 medium with L-glutamine (Thermo Fisher Scientific™), 10% fetal bovine serum (FBS) (Merck KgaA), and 1% penicillin/streptomycin (Thermo Fisher Scientific™), according to the manufacturer’s instructions. The medium for SeAx was supplemented with Il-2 (200 U/mL) (Merck KgaA). HEK293T cells were cultured in Dulbecco’s Modified Eagle Medium (DMEM) (Lonza) with 10% FBS (Merck KgaA) and 1% penicillin/streptomycin (Thermo Fisher Scientific™).

### 2.2 Clinical samples

Two SS blood samples: P1 (F, age 74), P2 (F, age 64), 1 MF blood sample with blood involvement: P3 (M, age 76) and one blood sample from MF patient without blood involvement P4 (M, age 62) were included in the study (Supplementary Table 1). CTCL samples were received from the Department of Dermatology, Poznań University of Medical Sciences in Poznan, Poland, and the Department of Dermatology, Venereology and Allergology, Medical University of Gdańsk, Poland. The study was approved by the Local Ethics Committee (Decision 629/20) and performed in accordance with the Declaration of Helsinki. Informed consent was obtained from all participants of the study. Samples from healthy donors (buffy coats; n = 4): HD1 (M, age 52), HD2 (M, age 28), HD3 (M, age 37), HD4 (M, age 27) were collected from the Regional Blood Donation and Blood Treatment Center in Poznań based on an agreement between RBDC and IHG PAS. Mononuclear cells from blood samples were purified using Histopaque. Subsequently, CD4 + lymphocytes were separated using the Human CD4+ T Cell Enrichment Kit (StemCell Technologies) based on negative selection. CD4+ T-cells were expanded and activated using Dynabeads Human T-Activator CD3/CD28 (Thermo Fisher Scientific). Primary T-cells were cultured in TexMACS Medium (Miltenyi Biotec) supplemented with 1% penicillin/streptomycin (Life Technologies) and IL2 (30 U/mL) (Miltenyi Biotec).

### 2.3 Generation of cells with HDAC10 overexpression or knockdown

In brief, lentiviral vectors for HDAC10 knockdown and overexpression were co-transfected with third-generation packaging plasmids—pMSCV-VSV-G, pRSV.REV, and pMDL-gPRRE into HEK293T cells using Lipofectamine 2000 (Thermo Fisher Scientific™). HEK293T cells (DSMZ ACC 635) were cultured in DMEM (Lonza) supplemented with 10% FBS (Sigma Aldrich) and 1% penicillin/streptomycin (Life Technologies). 24 h post-transfection, the medium was replaced. 48 and 72 h post-transfection, viral supernatant was collected, sterile filtered through a 0.45 um syringe filter, and stored at −80°C. Virus supernatant was added to cells together with polybrene (4 μg/mL). To establish a pure population of cells, selection with puromycin was performed for 5–7 days (2 μg/mL). The efficiency of the transduction was measured by flow cytometry regarding the green fluorescent protein (GPF) signal. Cells were harvested for RNA and protein. For HDAC10 knockdown miRZIP, KLHL6 plasmid was used. Three short hairpins RNAs were designed using the Broad Institute bioinformatic tool (https://portals.broadinstitute.org/gpp/public/seq/search) to knock down HDAC10 (shRNA1 on exon 4, sense: GAT​CCG​TGT​TCA​ACA​ACG​TGG​CCA​TAT​TCA​AGA​GAT​ATG​GCC​ACG​TTG​TTG​AAC​ACT​TTT​TG, anti-sense: AAT​TCA​AAA​AGT​GTT​CAA​CAA​CGT​GGC​CAT​ATC​TCT​TGA​ATA​TGG​CCA​CGT​TGT​TGA​ACA​CG; shRNA2 on exon 15, sense: GAT​CCG​AGG​AGT​CTG​TGG​CTG​AAC​ATT​TCA​AGA​GAA​TGT​TCA​GCC​ACA​GAC​TCC​TCT​TTT​TG, anti-sense: AAT​TCA​AAA​AGA​GGA​GTC​TGT​GGC​TGA​ACA​TTC​TCT​TGA​AAT​GTT​CAG​CCA​CAG​ACT​CCT​CG; shRNA3 on exon 7, sense: GAT​CCG​ATG​GGA​AAC​GCT​GAC​TAC​GTT​CAA​GAG​ACG​TAG​TCA​GCG​TTT​CCC​ATC​TTT​TTG, anti-sense: AAT​TCA​AAA​AGA​TGG​GAA​ACG​CTG​ACT​ACG​TCT​CTT​GAA​CGT​AGT​CAG​CGT​TTC​CCA​TCG). Control NT2 and SCR vectors were a kind gift from Prof. Anke van den Berg and Dr. Joost Kluiver (Yuan et al., 2018). For HDAC10 overexpression the following vectors designed by Vector Builder (Chicago, United States) were used: the lentiviral vector with HDAC10 ORF (pLV [Exp]; -EGFP:T2A:PurohPGK > hHDAC10 [NM_032019.6]/3xFLAG, ID: VB210105-1094shw), and the empty vector as a control (pLV [Exp]-CMV > EGFP (ns):T2A:Puro, ID: VB181012-1110yzy) (Supplementary Figure 1).

### 2.4 Immunofluorescence

Immunofluorescence was performed according to the standard protocol. Cells were fixed (4% formaldehyde/PBS pH 7,4), then permeabilized (0.1% Triton X-100/PBS) and any nonspecific binding sites blocked with 10% normal goat serum/PBS with Triton. Cells were incubated in the humidified chamber with primary antibody diluted in blocking solution (anti-HDAC10, H3413, Sigma Aldrich) and then with fluorescence coupled secondary antibody (Goat Anti Rabbit IgG H&L Alexa Fluor 488, ab150077, Abcam). Subsequently, a drop of antifade mounting medium with DAPI (Fluoroshield™ with DAPI; Sigma Aldrich) was added. Samples were analyzed using a Leica DMI8 laser-scanning confocal microscope (Leica Microsystems) and imaged processed using Leica Application Suite X software (Leica Microsystems).

### 2.5 Cellular fractionation

Cellular fractionation was performed as described previously (Winkle et al., 2022) with the use of 10 × 10^6^ cells. Buffers were supplemented with DTT, 1× EDTA-free protease inhibitors, and 20 U/mL RNaseOUT (when specified) immediately prior to use and maintained on ice. Cell pellets were resuspended in 250 µL Buffer W (300 mM sucrose, 10 mM Tris-HCl pH 8.0, 10 mM NaCl, 2 mM MgAc2, 0.5 mM DTT), followed by the addition of 250 µL Buffer L (Buffer W supplemented with 6 mM CaCl_2_, 0.2% IGEPAL CA-630, 0.5 mM DTT). Lysates were centrifuged at 1,000 × g, 4°C for 5 min to separate the cytoplasmic and nuclear fractions. The supernatant, representing the cytoplasmic fraction, was retained, and the nuclear pellet was processed further. Nuclei were resuspended in 500 µL Buffer G (50 mM Tris-HCl pH 8.0, 25% glycerol, 5 mM MgAc2, 0.1 mM EDTA, 5 mM DTT) with the addition of 500 µL Buffer U (1 M urea, 20 mM HEPES pH 7.5, 7.5 mM MgCl_2_, 0.1 mM EGTA, 300 mM NaCl, 1 mM DTT). The lysates were vortexed and centrifuged at 20,000 × g, 4°C for 10 min, and the resulting supernatant was collected as the nuclear fraction. The chromatin pellet was washed twice with Chromatin Wash Buffer (50 mM Tris-HCl pH 8.0 supplemented with 20 U/mL RNaseOUT) and subjected to sonication in 300 µL RIPA buffer (Merck KgaA) using a Bioruptor Pico (Diagenode) for three cycles of 20 s ON, 30 s OFF. To ensure the accuracy of the cellular fractionation process, Western Blot was performed with specific antibodies targeting established markers for different subcellular fractions were used –anti-beta-Tubulin (cytoplasmic) (Abcam, ab18207, 1:5000), anti-U1snRNP (nuclear) (Santa Cruz Biotechnology, sc-390899, 1:1500), anti-Histone H3 (chromatin) (Abcam ab18521, 1:500).

### 2.6 Western blot

**FIGURE 1:**
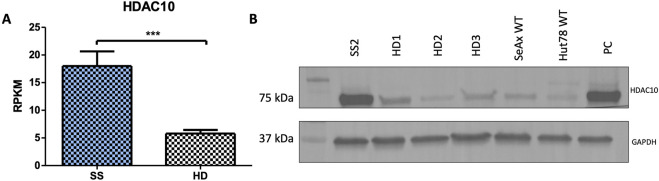
HDAC10 expression in Sézary syndrome. **(A)** RPKM values for the *HDAC10* gene calculated from RNAseq data (SS n = 9, HD n = 10 (Iżykowska et al., 2017)). Data expressed as the mean ± SEM, Student’s t-test (two-tailed), ***p = 0.0002. **(B)** Western blot analysis of HDAC10 in Sezary syndrome (SS2) patient, healthy donors (HD1-3), and Sezary syndrome cell lines. PC–positive control (SeAx with HDAC10 overexpression); RPKM (Reads Per Kilobase Million).

**FIGURE 2:**
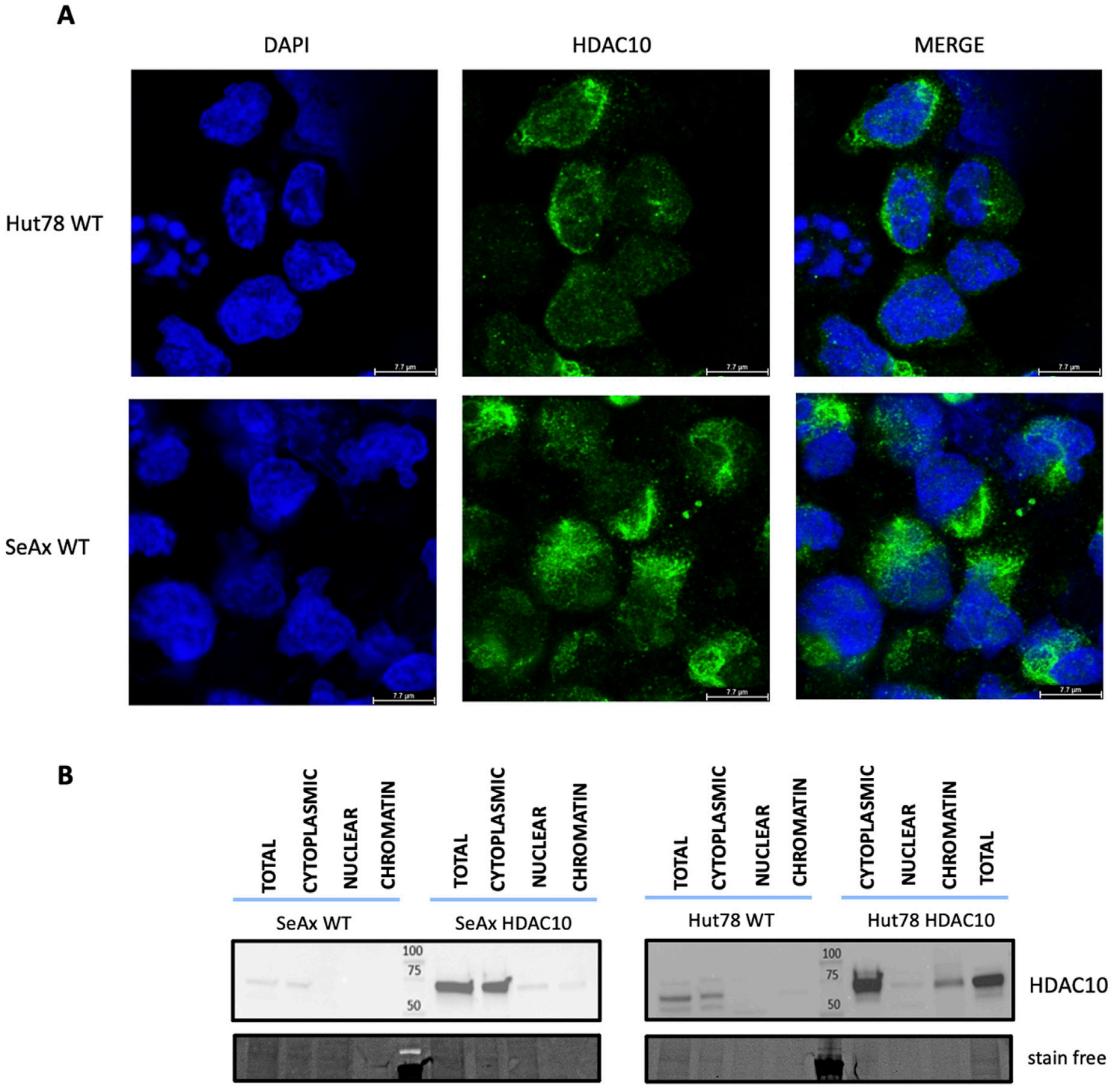
Localization of HDAC10 in CTCL cell lines. **(A)** Immunofluorescence analysis of HDAC10 localization in SeAx and Hut78 cell lines using a specific anti-HDAC10 antibody. Representative images show the predominant localization of HDAC10 in the cytoplasm. **(B)** Cellular fractionation of CTCL cells assessed by Western blot, illustrating the distribution of HDAC10 in different cellular fractions–cytoplasmic, nuclear and chromatin.

Whole-cell lysates were prepared from 5–10 × 10^6^ cells. The cells were washed with 1X PBS and lysed in RIPA buffer (Merck KgaA) containing 1X protease inhibitor cocktail (Bioshop Canada Inc.) for 30 min on ice. The samples were then sonicated (3 cycles, ON 20 s, OFF 30 s, Biorupto Pico, Diagenode). Total protein concentration in cell extracts was measured using Pierce BCA protein Quantification kit (Thermo Fisher Scientific™) and samples were stored at −80°C until assayed. Before being loaded onto the gel, the samples were heated at 95°C for 5 min in the heating block. Protein extracts were then combined with Sigma’s Laemmli 4X sample buffer, denatured, and separated on Mini-PROTEAN Stain-free gels (Bio-Rad) using a Mini-PROTEAN^®^ Tetra electrophoresis instrument (Bio-Rad). After electrophoresis, proteins were transferred to PVDF membranes using the Mini Trans-Blot^®^ cell system (Bio-Rad). Membranes were blocked and then incubated with rabbit monoclonal anti-HDAC10 antibody (H3413, 1:10,000, Sigma Aldrich). After incubation with primary antibodies, membranes were treated with HRP-conjugated secondary antibody (ab6721, 1:3000, Abcam). Protein bands were visualized by chemiluminescence using Clarity Western ECL Substrate (Bio-Rad) and detected by ChemiDoc™ Imaging Systems (Bio-Rad). Quantitative Western blot analysis was performed using ImageLab™ software. Results were normalized using reference gene GAPDH (sc-365062, Santa Cruz Biotechnology) (Figure 1B) or stain-free technique (Figures 2B, 6D; Supplementary Figures 2A, B, 4A–D, 8A, B) that quantifies total protein directly on the membrane.

### 2.7 RNA extraction

Total RNA was extracted from the cells using the Quick-RNA Miniprep Kit (Zymo Research) according to the manufacturer’s instructions. RNA quantity was measured with NanoDrop 2000 (Thermo Fisher Scientific Inc.) and RNA integrity was analyzed using the Agilent High Sensitivity RNA ScreenTape System (Agilent Technologies). RIN scores for all samples were above 9.

### 2.8 Reverse transcription-quantitative polymerase chain reaction (RT-qPCR)

cDNA was synthesized from 0.5 µg of RNA using SuperScript™ IV Reverse Transcriptase according to the manufacturer’s protocol. Following reverse transcription, qPCR was performed using the 5 x HOT FIREPol^®^ Probe qPCR Supermix (Solis Biodyne) and TaqMan Gene Expression Assays (Applied Biosystems). The expression of HDAC10 was examined with Hs00368899_m1 assays and normalized to beta-2 microglobulin (B2M) (Hs00984230_m1) reference gene mRNA level. RT-qPCR was perfomed using a CFX96 real-time system (Bio-Rad). Each sample was tested in triplicate, and the median value was used to determine relative gene expression using the Livak method (2^−Δ*CT*^).

### 2.9 Library preparation, next-generation sequencing, and bioinformatic analysis

The RNAseq service with library preparation, next-generation sequencing, and bioinformatics analysis was performed by Macrogen-Europe B. V. (Amsterdam, Netherlands). RNA samples (at least 1 µg in at least 20 μL, RIN, higher than 7.0, rRNA ratio at least 1.0) were subjected to input material quality and quantity examination. The library was prepared using TruSeq mRNA stranded kit (poly-A selection) (Illumina). Sequencing was performed on the NovaSeq6000 Platform at the pair-end configuration (2 × 150 bp) and the throughput of 60M total reads per sample. The bioinformatics analysis including standard mapping, expression profiling, DEG analysis and functional annotation using GO and KEGG database was provided by Macrogen Europe B.V. The RNAseq data were submitted to the Gene Expression Omnibus database (GSE273693).

### 2.10 Validation of RNAseq data by RT-qPCR

cDNA was synthesized from 0.5 µg of RNA using SuperScript™ IV Reverse Transcriptase according to the manufacturer’s protocol. Following reverse transcription, qPCR was performed using the 5 x HOT FIREPol^®^ EvaGreen^®^ qPCR Supermix (Solis Biodyne) and specific for chosen genes primer pairs (ACVR1C: Forward: AAA​ACC​GAA​TGC​TGC​TTC​AC; Reverse: GTC​CAA​GTT​TTG​GGG​CAT​TT). The PCR reactions were conducted on the CFX96 Touch Real-Time PCR Detection System (Bio-Rad, Hercules, CA, United States). Relative expression levels were calculated using the 2^−Δ*CT*^ formula, with the reference genes B2M in SeAx and primary samples, and GAPDH in Hut78 employed for the normalization of gene expression. cDNA from three SS patients (SS2, and two: SS16-17 from previous study (Rassek et al., 2023a)), and three cDNA from healthy donors (HD1-HD3) was used for validation.

### 2.11 GFP growth competition assay

SeAx and Hut78 cells were transduced with miRZIP lentivirus, achieving an infection efficiency of 50%. The proportion of GFP-positive cells was assessed by flow cytometry (CytoFLEX S flow cytometer, Beckman Coulter) on day 4 after transduction and then monitored three times a week for 3 weeks. Data were analyzed using Kaluza Analysis Software (Beckman Coulter). To assess the effect of HDAC10 knockdown on cell proliferation, the percentage of GFP-positive cells on day 6 was normalized to 100% and the fold change from this baseline was calculated for each subsequent time point. Statistical significance in GFP assays was determined using mixed model analysis as previously described (Yuan et al., 2018).

### 2.12 Apoptosis, cell cycle and autophagy functional assays

The percentages of apoptotic cells were determined in transduced cells harvested on days 7 (SeAx), 12 (Hut78), and 8 (primary CD4^+^ T-cells) after transduction. Briefly, cells were washed twice with cold 1X PBS and resuspended at a concentration of 1 × 10^6^ cells/mL in 1X Binding Buffer. Cells were stained with Annexin V/APC and 7AAD according to the manufacturer’s protocol (BD Biosciences) and analyzed using flow cytometry (CytoFLEX S Flow Cytometer, Beckman Coulter). BrdU staining (APC/BrdU Flow Kit, BD PharmigenTM) and flow cytometry analysis was applied to study cell cycle. Briefly, 0,5 × 10^6^ cells were cultured and treated with 10–20 µM bromodeoxyuridine (BrdU) to incorporate it into the newly synthesized DNA, according to manufacturer’s protocol. Cells were blocked and incubated with anti-BrdU primary antibody followed by fluorophore-conjugated secondary antibody. Autophagy was measured using the autophagic flux assay by WB and Anti-LC3-I/II Antibody (Merck ABC929). Briefly, 2 × 10^6^ cells with HDAC10 knockdown and non-targeting control were cultured on 6-well plates for 24 h with the following compounds: 0.5 nM Rapamycin (Symbiosis), 100 µM Chloroquine diphosphate (Symbiosis), and after that whole-cell protein lysates were prepared, the protein concentration was measured and analyzed using Western blot, as described before.

### 2.13 Compounds, IC50, and combination index (CI)

Selective HDAC10 inhibitor DKFZ-748 was a kind gift from Dr. Aubry K. Miller and Raphael Steimbach from the DKFZ German Cancer Research Center (Heidelberg, Germany) (Steimbach et al., 2022). DKFZ-748, and Camptothecin (Sigma Aldrich) were diluted in DMSO at the concentration of 100 mM and 1 mM, respectively. The working solutions for both compounds were prepared using the corresponding volumes of cell medium. The effect of the DKFZ-748 and Camptothecin on cell survival was measured with CellTiter 96^®^ AQueous One Solution Cell Proliferation Assay (MTS) (Promega) and the ELx808 Biotek Plate Reader. Cells were cultured in the following concentrations of DKFZ-748: primary cells: 100 μM, 50 μM, 25 μM, 12.5 μM, 6.25 μM, 3.13 μM, and 1.56 μM; CTCL cell lines: 400 μM, 200 μM, 100 μM, 50 μM, 25 μM, 12.5 μM, 6.25 μM and 3.13 μM. For the Camptothecin the following concentrations were used: CTCL cell lines 20 nM, 10 nM, 5 nM, 2.5 nM, 1.25 nM, 0.625 nM, 0.312 nM, and 0.156 nM. To study the synergy 3 concentrations were used, low, medium, and high. The absorbance was measured after 48 h and the IC50 (half-maximal inhibitory) values were calculated in GraphPad Prism. The Combination index values were calculated by using the Chou-Talalay method. Experiments were performed in at least two biological replicates, each with three technical replicates.

### 2.14 Metabolic activity (resazurin) assay

0.05 × 10^6^ cells were incubated with increasing concentrations (0, 0.001, 0.01, 0.1, 1, 10 µM) of venetoclax and navitoclax (Selleckchem) for 72 h. AlamarBlue (Thermo Fisher Scientific) was added 8 hours prior to read-out (extinction 560 nm, emission 590 nm). Metabolically active cells were calculated in percentage based on the untreated cells. Experiments were performed 3 times and each experiment was performed in quadruplicate.

### 2.15 BH3 profiling

BH3 profiles were acquired by a plate-based assay using JC-1 dye (Ryan and Letai, 2013). Peptide concentrations used for profiling were 10 µM PUMA2A, 1 and 10 µM BIM, 10 μM HRK, 10 µM NOXA, 0.1, 1 and 10 µM BAD. A concentration of 5 µM was used of carbonyl cyanide-p-trifluoromethoxyphenyl hydrazone (FCCP) as a positive control. Fluorescence was measured every 5 min for 2 h at excitation 545 nm and emission 590 nm. The percentage of mitochondrial outer membrane permeabilization (MOMP) was calculated using PUMA2A as a negative and FCCP as a positive control using the area under the curve (1-((AUC sample- AUC FCCP)/(AUC PUMA2A-AUC FCCP))x100. The ΔMOMP was calculated by subtracting the MOMP of HUT78 HDAC10 of the MOMP of HUT78 GFP. A ΔMOMP ≥ 20% is considered biologically relevant (de Jong et al., 2019).

### 2.16 Statistical analysis

Data were analyzed with GraphPad Prism and Microsoft Excel. For two independent groups, the t-tests were used while one-way ANOVA was used for 3 or more groups. Values were considered significant at p < 0.05.

## 3 Results

### 3.1 HDAC10 is overexpressed in Sézary Syndrome Patients

In addition to the existing literature and studies on HDAC inhibitors in CTCL, previous next-generation sequencing (NGS) analysis of SS samples performed by Iżykowska et al. suggested the importance of HDAC10 in SS pathogenesis. Retrospective RNAseq data showed HDAC10 to be overexpressed in SS patients compared to healthy controls (Figure 1A) (Iżykowska et al., 2017), and this observation was confirmed on protein level using Western blot (Figure 1B). These findings prompted us to further investigate the role of HDAC10 in the context of the molecular background of SS.

### 3.2 HDAC10 protein is mainly localised in the cytoplasm

**FIGURE 3:**
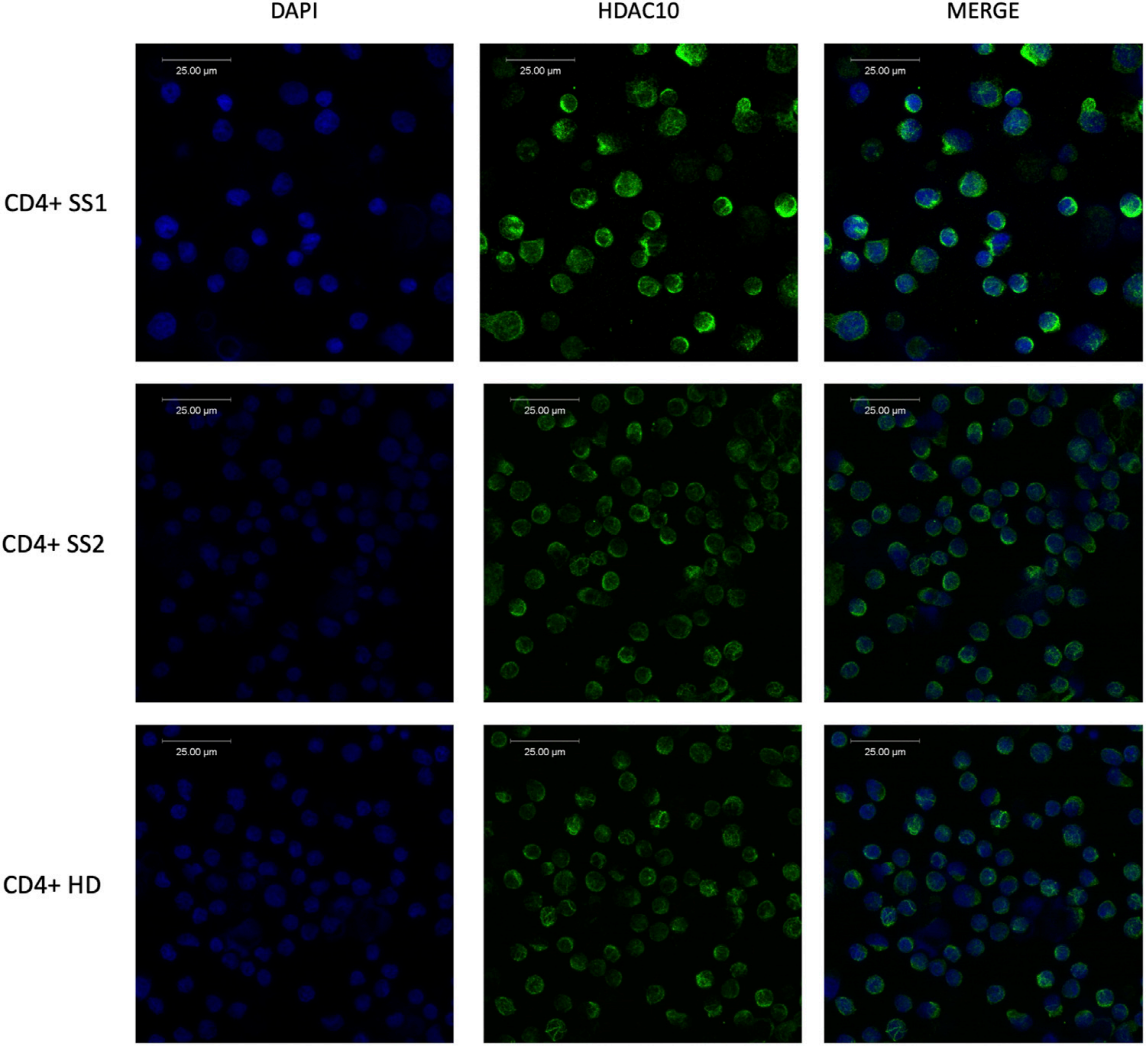
Immunofluorescence analysis of HDAC10 in primary cells from patients with Sézary syndrome (SS) and healthy donor (HD) using specific anti-HDAC10 antibodies. Cytoplasmic localization of HDAC10.

Class II HDAC proteins, including HDAC10, can be localized both in the nucleus and in the cytoplasm. HDAC10 localization in our study was examined using a dual approach including immunofluorescence staining and cellular fractionation. Immunofluorescence analysis in cell lines (Figure 2A) and primary cells (Figure 3), including T-cells from SS1, SS2, and HD3, revealed mainly cytoplasmatic localization of HDAC10. This observation was confirmed by Western blot analysis of fractionated samples from CTCL cell lines (SeAx and Hut78 - Figure 2B), both wild type and with induced HDAC10 overexpression (Supplementary Figure 2). The accuracy of the cellular fractionation process was guaranteed by using the specific antibodies targeting established markers for different subcellular fractions (Supplementary Figure 3). In HDAC10-overexpressing cells not only a cytoplasmatic but also a nuclear localization of HDAC10 was visible (Figure 2B). This data shows that although the majority of HDAC10 protein is localized in the cytoplasm, there is a fraction present in the nucleus, and the localization is not changed in cancer cells compared to controls (Figure 2B) or does not change drastically in time (Supplementary Figure 4).

### 3.3 HDAC10 overexpression modulates apoptosis responses and BH3 profiling

**FIGURE 4:**
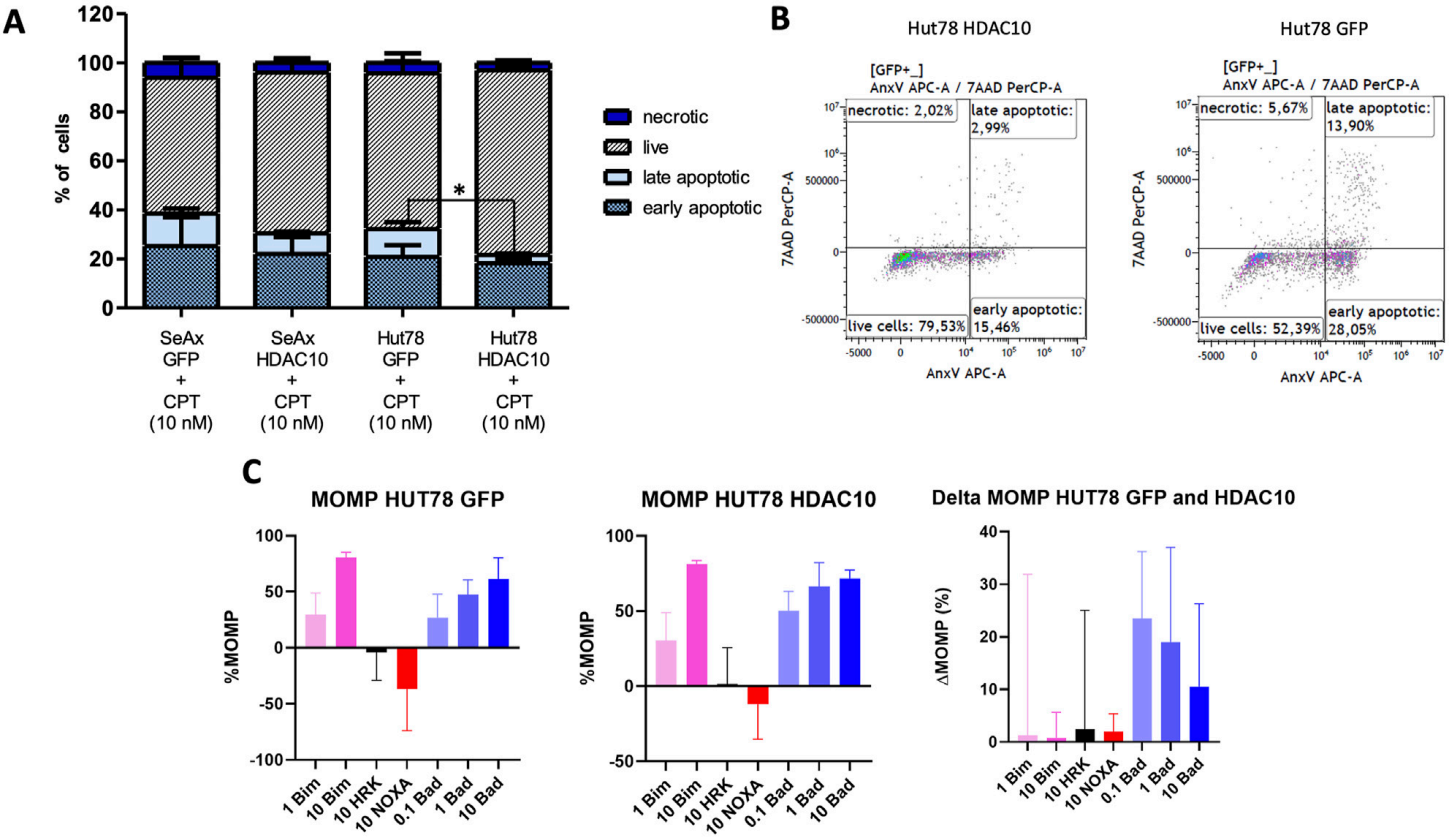
Apoptotic response in SeAx and Hut78 cells overexpressing HDAC10 and BH3 profiling analysis. **(A)** Apoptosis analysis in HDAC10 overexpressing SeAx and Hut78 cells, compared to controls. The percentages of live, early apoptotic, late apoptotic, and necrotic cells were determined using flow cytometry analysis with Annexin V/7-AAD staining. Data expressed as the mean ± SEM of 4 independent experiments, Student’s t-test (two-tailed), *p < 0.05. CPT-Campthothecin. **(B)** Representative flow cytometry analysis plots of Hut78 with HDAC10 overexpression and control **(C)** BH3 profiling analysis of Hut78 cells upon specific peptides.

A series of functional assays was performed to determine the role of HDAC10 in Sézary syndrome cell lines. Overexpression of HDAC10 alone did not significantly affect cellular processes such as cell proliferation, cell cycle progression, or apoptosis (Supplementary Figure 5). However, after exposure to the pro-apoptotic compound Camptothecin (CPT, 10 nM, 48 h incubation time), an inhibitory effect on apoptosis was observed in cells overexpressing HDAC10 (Figures 4A, B; Supplementary Figure 6). Based on the results of the apoptosis assay, we extended our study to include BH3 profiling. The BH3 profile of Hut78 GFP showed a response for BIM (at 1 µM 29% and at 10 µM 80%) which indicates that the cells were able to go in apoptosis. The MOMP for HRK and NOXA were detected negative and showed that the cells do not depend on BCL-XL (HRK) or MCL-1 (NOXA). The MOMP for BAD was increasing with the peptide concentration (at 0.1 µM 27%, at 1 µM 47% and at 10 µM 61%), indicating that the cells were primed and dependent on BCL-2 or BCL-W. The BH3 profile for Hut78 with HDAC10 overexpression was similar to the profile of Hut78 GFP (Figure 4C), although responses have increased for BAD to 50%, 66% and 72%. In the ΔMOMP the effect of HDAC10 overexpression in Hut78 became clear with the increase in BAD sensitivity of 23, 19% and 11%, showing a consistent increase in BCL-2 or BCL-W dependence. Since an increase of more than 20% is considered biologically relevant, we tested if Hut78 HDAC10 has increased sensitivity to BCL-2 inhibitor venetoclax and BCL-2/BCL-XL/BCL-W inhibitor navitoclax (Supplementary Figure 7). The sensitivity to venetoclax and navitoclax was not changed in Hut78 after the overexpression of HDAC10 with an IC50 for venetoclax of 12 and 16 µM for venetoclax and 0.5 and 0.8 µM for navitoclax.

### 3.4 HDAC10 gene silencing results in growth inhibition and induction of apoptotic and autophagic processes

**FIGURE 5:**
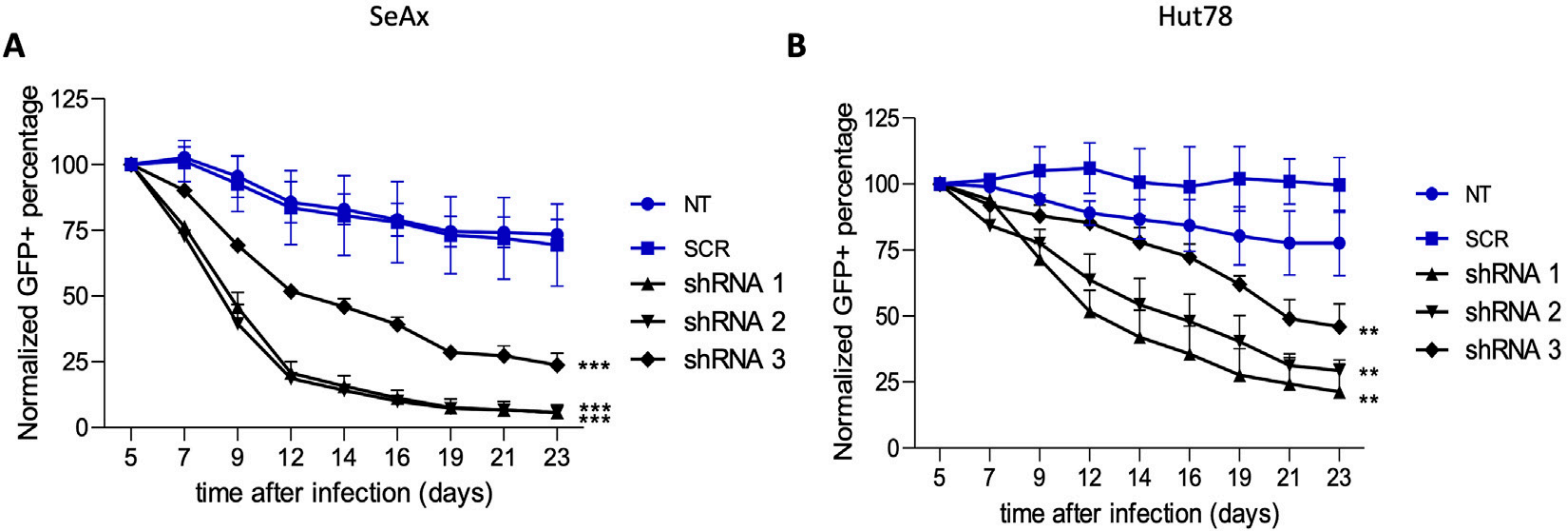
Green fluorescent protein (GFP) growth competition assay with specific shRNAs targeting HDAC10 in **(A)** SeAx and **(B)** Hut78 cell line. The effect on HDAC10 knockdown on cell growth was assessed by following percentage of GFP+ cells for 23 days post-transduction, with the GFP percentage normalised to day 5 (n = 3); ***p < 0.001, **p < 0.01. NT-non-targeting, SCR–scrambled.

To establish the function of HDAC10 in malignant cells, we analyzed the effect of HDAC10 gene silencing in SeAx and Hut 78 cell lines, both of which express endogenous HDAC10. The efficacy of shRNA targeting the HDAC10 gene was confirmed in both cell lines (Supplementary Figure 8). Knock-down of HDAC10 resulted in a marked inhibitory effect on cell growth (Figure 5). Fourteen days after transduction, the percentage of GFP-positive cells in SeAx cell lines transduced with HDAC10-specific shRNA was reduced by more than 50% compared to non-targeted (NT) and scrambled (SCR) controls. The inhibitory effect on SeAx cell growth was more pronounced in the case of shRNA1 and shRNA2. In Hut 78 cell lines, a similar decrease of more than 50% in GFP-positive cells was observed 21 days after transduction, with growth inhibition being also stronger for shRNA1 and shRNA2.

**FIGURE 6:**
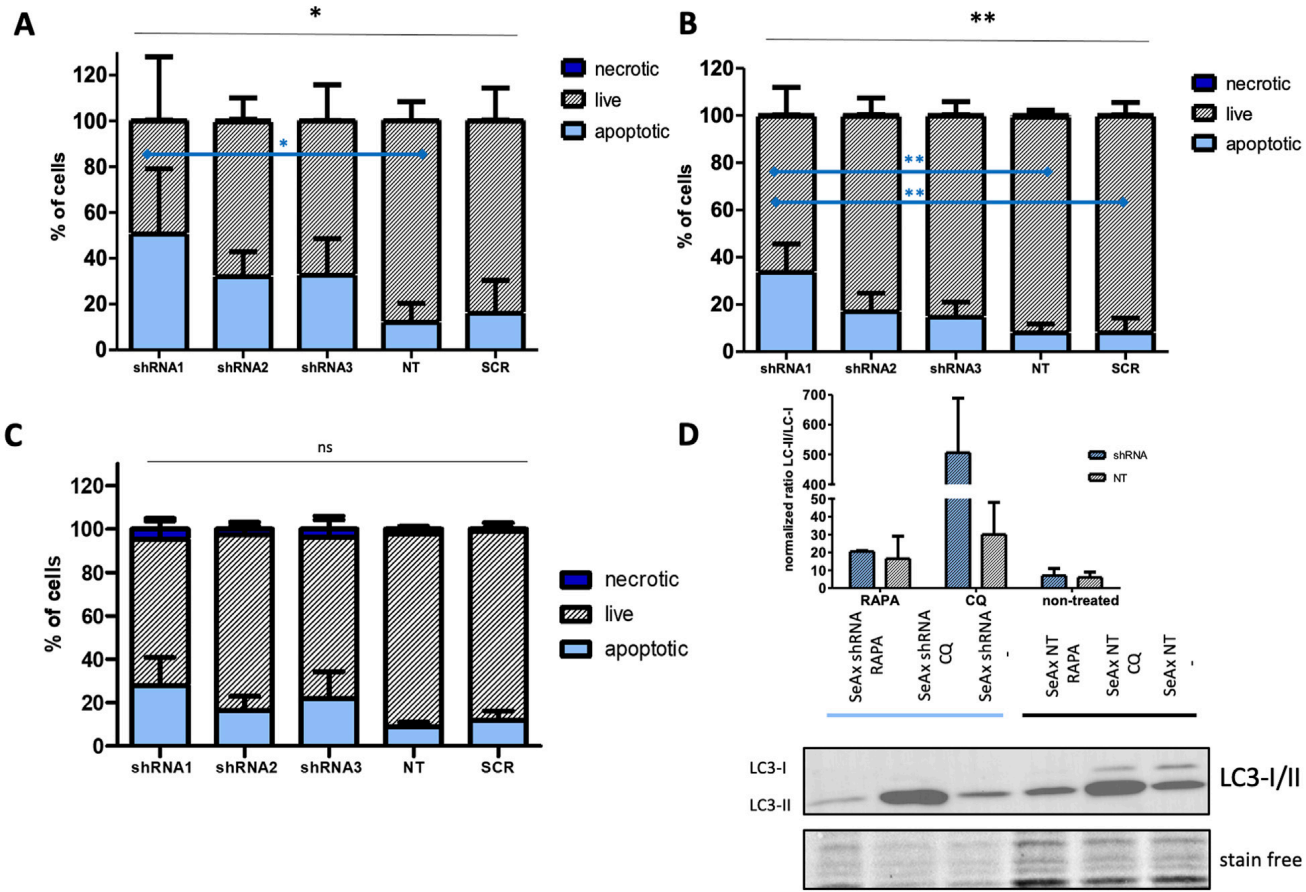
HDAC10 gene silencing induces apoptotic and autophagic processes in SS cell lines. **(A, B)** Flow cytometric analysis of apoptosis in SS cell lines after HDAC10 knockdown using Annexin V/7 AAD staining in **(A)** SeAx (day 7 post-transduction) and **(B)** Hut78 cell line (day 12 post-transduction). Data expressed as the mean ± SEM (n = 4), One-way ANOVA, *, **p < 0.05 (black lines); Tukey Test, *, **p < 0.05 (blue lines) The results indicate an increased level of early and late apoptotic cells compared to the control. **(C)** Flow cytometry analysis of apoptosis in CD4^+^ T-cells from healthy donors with HDAC10 knockdown. Data expressed as the mean ± SEM (n = 3), One-way ANOVA, ns–not significant. The results indicate an increased level of early and late apoptotic cells compared to the control **(D)** Western blot analysis of autophagy in SeAx cell line after HDAC10 knockdown. RAPA–rapamycin, CQ-chloroquine.

Further research focused on apoptosis and autophagy processes that were performed to provide a comprehensive understanding of the mechanisms underlying the growth inhibition observed upon HDAC10 knockdown in SS cell lines. Flow cytometry analysis of apoptosis demonstrated that HDAC10-specific shRNAs induced apoptosis in both cell lines compared to controls (Figures 6A, B). The mean percentage of apoptotic cells in all three knockdown cell lines (sh1, sh2, sh3) in SeAx was 38.39% versus 14.02% in both controls (SCR, NT2) (p = 0.0060), and 21.69% versus 8.007% (p = 0.0113) in Hut78, respectively. The time points for analysis were determined based on the GFP growth competition assay, which identified day seventh as optimal for the SeAx cell line and day 12th for the Hut 78 cell line. The same analysis was performed in healthy primary T-cells (Figure 6C), on day 9 after transduction, also showing the pro-apoptotic effect of HDAC10 knockdown on cells (22.05% vs. 10.33%, p = 0.246).

In the autophagy analysis using Western blot (Figure 6D), SS cells were treated with rapamycin (RAPA), an autophagy inducer that activates the process by repressing mTORC1, and chloroquine (CQ), which inhibits autophagosome fusion with lysosomes and slows down lysosomal acidification. Western blot analysis measured the ratio of LC3-II to LC3-I to quantify autophagic flux, where the ratio increase indicates autophagy induction, while a decrease suggests inhibition or impairment of autophagy. In the assay, SeAx cells treated with RAPA and CQ were analyzed. Notably, CQ-treated cells exhibited a significant increase in the LC3-II to LC3-I ratio, indicating a substantial accumulation of autophagosomes due to inhibited autophagosome-lysosome fusion upon HDAC10 silencing. This accumulation reflects increased autophagic activity in response to a lack of HDAC10.

### 3.5 Inhibition of HDAC10 with selective HDAC10i indicated increased sensitivity to Camptothecin

**FIGURE 7:**
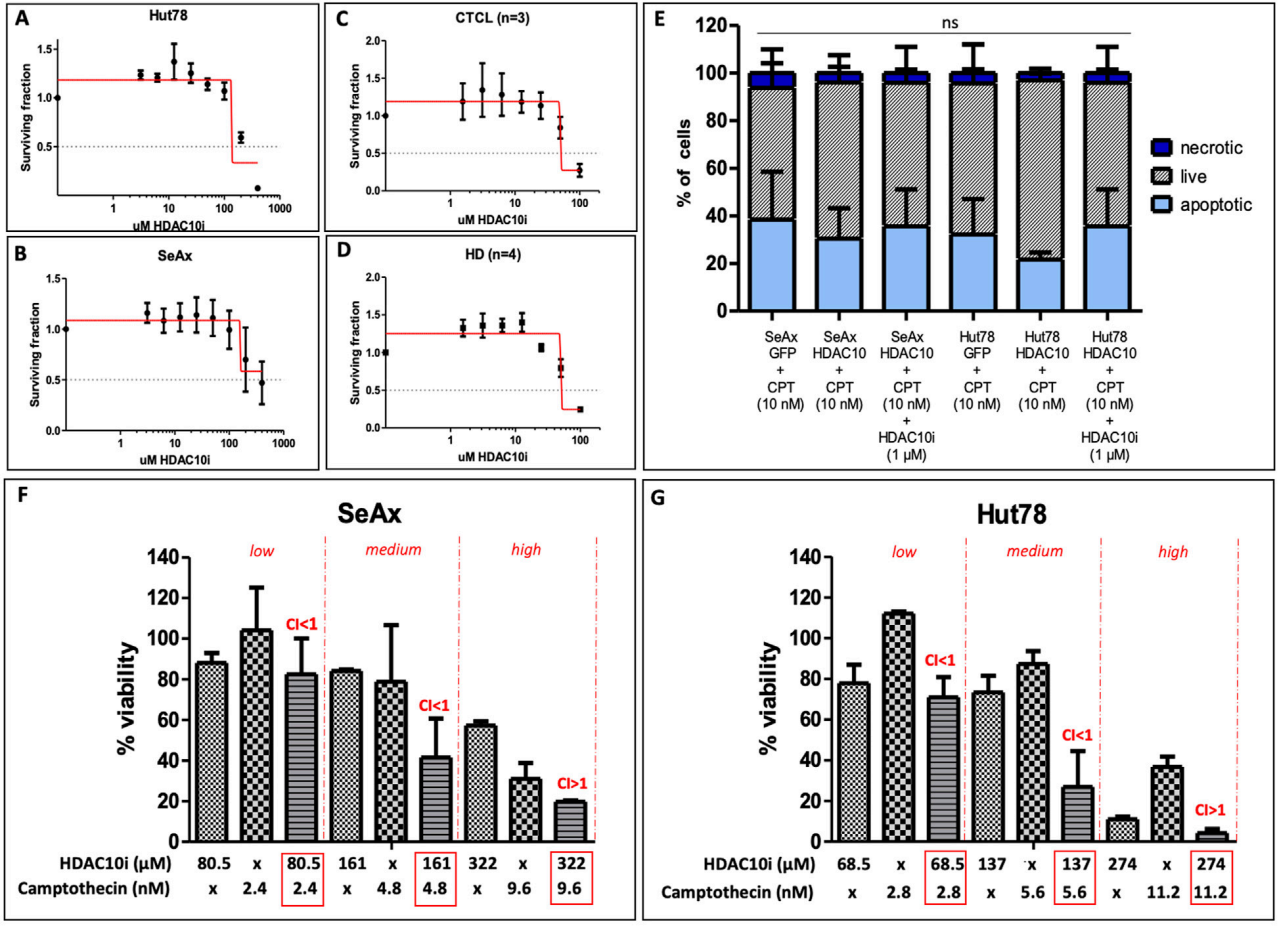
Effect of selective HDAC10 inhibition with DKFZ-748 on CTCL cell lines and primary cells. **(A)** IC50 values calculated for Hut78 cell line, an experiment performed in three biological and three technical replicates, IC50 = 137 μM; **(B)** IC50 values calculated for SeAx cell line, an experiment performed in three biological and three technical replicates, IC50 = 161 μM; **(C)** IC50 values calculated for a group of CTCL patients (n = 3), an experiment for each patient performed in three technical replicates (median values calculated), IC50 = 50 μM; **(D)** IC50 values calculated for a group of HD (n = 4), an experiment for each patient performed in three technical replicates (median values calculated), IC50 = 50 μM; **(E)** apoptosis analysis in SeAx and Hut78 cells with HDAC10 overexpression incubated with Camptothecin alone (10 nM) and in the presence of HDAC10i (1 µM); Data expressed as the mean ± SEM (n = 4), One-way ANOVA, ns; **(F)** Combination index (CI) for Camptothecin and HDA10i in SeAx; **(G)** CI for Camptothecin and HDAC10i in Hut78; CI < 1 = synergy; CI > 1 = antagonism.

The cytotoxic effect of a novel, specific to HDAC10 inhibitor (DKFZ-748) (Steimbach et al., 2022) was tested in CTCL cell lines and primary cells from CTCL patients, including SS2, MF1, and MF2 and in healthy donors (HD1-4). To examine the cytotoxicity of inhibitors the IC50 values were calculated. For both CTCL cell lines, Hut78 and SeAx, there was no cytotoxicity observed below the concentration of 100 μM, the IC50 was approximately 137 µM and 161 μM, respectively (Figures 7A, B). Primary cells were more sensitive to the selective HDAC10i, however, no difference was observed between the T-cells from CTCL patients and lymphocytes from healthy donors (Figures 7C, D), as the IC50 value for both groups was the same (50 µM). To further explore the possible effect of HDAC10i we repeated the apoptosis analysis on SeAx and Hut78 cells with HDAC10 overexpression, in the presence of Camptothecin (10 nM), and additionally in the presence of Camptothecin together with HDAC10i (1 µM). The experiment confirmed the previous observation, that there was a slight decrease in the apoptosis progression in cells with HDAC10 overexpression, and this effect was reversed in the presence of HDAC10i (Figure 7E). In the following experiments, the possible synergistic effect of HDAC10i with the pro-apoptotic drug camptothecin was studied. The series of experiments were performed with three different combinations of drug concentrations referred to as low, medium, and high, calculated relative to IC50 values for both compounds (Figures 7F, G; Supplementary Figure 9). The analysis showed indeed the synergistic effect of two compounds used in low (SeAx C1 = 0.45; Hut78 CI = 0.34) and medium (SeAx CI = 0.64; Hut78 CI = 0.55) concentrations (CI < 1). In contrast, for high (SeAx CI = 1.40; Hut78 CI = 1.59) concentrations the effect was antagonistic (CI > 1). Our experiments showed that there is a relatively low potential of using HDAC10i inhibitor in CTCL treatment alone due to its low cytotoxicity. However, there is a possibility of using this compound in synergistic combination with other proapoptotic drugs.

### 3.6 Identification of whole transcriptome alterations upon HDAC10 activity

**FIGURE 8:**
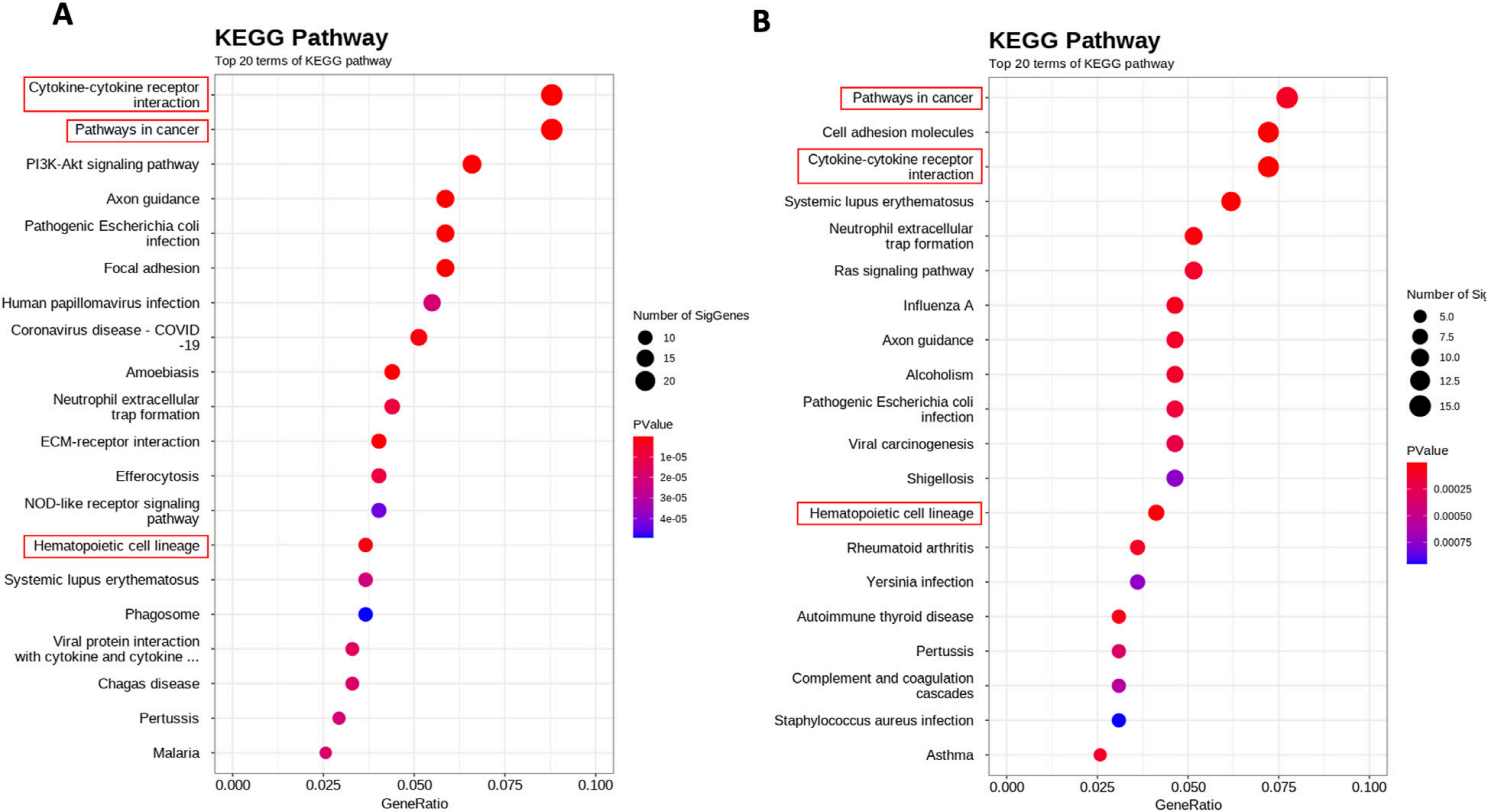
Whole transcriptome alterations upon HDAC10 knockdown and overexpression in SeAx cell line. Scatterplot of enriched KEGG pathways for DEGs between **(A)** SeAx HDAC10 knockdown/control **(B)** SeAx HDAC10 overexpression/control. The dot color represents the P value, and the dot size represents the number of genes enriched in the reference pathway.

The whole transcriptome sequencing of SS cell lines was performed for the identification of alterations in the global expression of genes and the modulation of cellular pathways under the influence of the HDAC10 (Figure 8).

**FIGURE 9:**
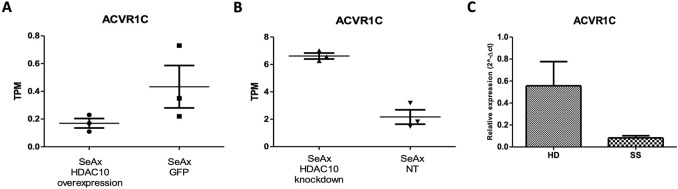
*ACVR1C* expression in: **(A)** SeAx cell line with HDAC10 overexpression (SeAx H10) and control cells transduced with empty vector (SeAx GFP) (RNAseq data; fold change = −2,33; raw. p = 0.005); **(B)** SeAx cell line with HDAC10 knockdown (SeAx H10 KD) and control cells transduced with non-targeting vector (SeAx NT2) (RNAseq data; fold change = 2,46; raw. p < 0.0001); TPM (Transcripts Per Kilobase Million); **(C)** RT-qPCR analysis of *ACVR1C* expression in Sézary syndrome patients (SS n = 3) and healthy donors (HD n = 3).

In the differential expression gene (DEG) analysis comparing SeAx with HDAC10 overexpression and SeAx with HDAC10 knockdown, 100 common genes were identified. From these, 29 genes were exhibiting opposite expression patterns between the overexpression and knockdown, including *CASP4, IL7R, CXCL8, SH2D2A, HAVCR2, ACVR1C, PGF,* and *DEPTOR* (Supplementary Figure 11; Supplementary Table 2). Downregulation of *ACVR1C* was confirmed in a group of SS patients (n = 3) compared to healthy donors (n = 3) (Figure 9; Supplementary Figure 10). Selected genes were identified to be involved in deregulated key signaling pathways, including cytokine-cytokine receptor interaction (*IL7R, CXCL8, ACVR1C*), hematopoietic cell lineage (*IL7R*), PI3K-Akt signaling pathway (*COL4A6, IL7R, PGF*), pathways in cancer (*IL7R, CXCL8, PGF*), Ras signaling pathway (*PGF*), MAPK signaling pathway (*PGF*), JAK-STAT signaling pathway (*IL7R*), transcriptional misregulation in cancer (*CXCL8*), and NOD-like receptor signaling pathway (*CASP4*). Altogether, 55 common KEEG pathways were identified to be altered in SeAx with HDAC10 overexpression and SeAx with HDAC10 knockdown (Supplementary Tables 4, 5).

In DEG analysis comparing SeAx with HDAC10 knockdown and Hut78 with HDAC10 knockdown, 43 genes in common were identified. From these, 32 genes had the same effect in both conditions. – including *IGLL1, MDK, SMC1A, AURKB, TJP2, C1D, RABL2B, RABL2A, EMC4, PIPOX, CIP2A, SLC35B4, PIGS* (Supplementary Figure 12; Supplementary Table 3). According to GO selected genes are involved in cell cycle (*SMC1A, AURKB*), apoptosis (*AURKB, EMC4)* and cell proliferation (*RABL2B).* Moreover, the analysis revealed 17 common dysregulated KEGG pathways (Supplementary Table 4), which include PI3K-Akt signaling pathway, pathways in cancer, and the MAPK signaling pathway. These findings highlight the potential role of HDAC10 in critical cellular processes and disease mechanisms in SS.

## 4 Discussion

In this study, we explored the role of HDAC10 in Sézary syndrome, a leukemic and very aggressive subtype of cutaneous T-cell lymphoma. SS is a very challenging condition, in both, scientific research and clinical practice, due to its aggressiveness, resistance to treatment, and also symptoms, which can mimic different dermatoses in the early stages, affecting the diagnosis timing, and by this the overall patient survival.

As in the last decades, the attention was promoted to histone deacetylase inhibitors, the HDACs started to be considered as potential molecular players in the pathogenesis of various diseases. Our research indicated that HDAC10 is overexpressed in SS patients. Based on this discovery, the current study specifically addresses the unexplored role of HDAC10. Our findings show that HDAC10 is primarily located in the cytoplasm of Sézary cells, which aligns with its known functions in various cellular processes such as cell cycle regulation, apoptosis, and autophagy. Cytoplasmatic localization may suggest its involvement in interactions with cytoplasmatic proteins. It is worth mentioning, that HDAC10 belongs to class IIb HDACs, together with different histone deacetylase–HDAC6, which is known to be predominantly localized also in the cytoplasm, but still regulates the activities of the transcription factors in the nucleus (Yang et al., 2015). Cytoplasmic HDAC6 has been shown to interact with proteins such as α-tubulin, cortactin, heat shock protein 90, β-catenin, and peroxiredoxins, playing a role in the regulation of apoptosis, tumor growth and migration (Yang et al., 2015). The cytoplasmic localization of HDAC10 observed in Sézary cells also provides intriguing insights into its potential functions beyond classical histone deacetylation. Additionally, cytoplasmic HDAC10 could participate in signaling cascades like those mediated by, for example, PI3K/AKT/mTOR, further highlighting its role in tumorigenesis. Further studies are needed to characterize its specific protein targets and their implications in cancer pathogenesis.

Genetic alterations in SS have been previously studied and revealed numerous changes, contributing to the complexity of the disease, and highlighting the heterogeneous nature of SS. Studies performed by Iżykowska et al. (2013, 2017) identified a variety of chromosomal rearrangements and copy number changes in Sézary syndrome affecting regions 6q23-27, 8q, 17p, 10q, 2p, 11q, and 9p, with altered expression of genes including, *EHD1, MTMR2, RNF123, TOX*, and *TMEM244* - which was further indicated to be crucial for CTCL cell growth (Rassek et al., 2023b). Other studies also revealed somatic mutations and deletions in tumor suppressor and regulatory genes, indicating increased activity of the MAPK, NFκB, and NFAT pathways (da Silva Almeida et al., 2015). Although published studies prompted the genetic landscape story of Sézary syndrome, the pathophysiology of the disease remains unclear. In the present study, we decided to explore what alterations in the whole-transcriptome of SS cells could be related to HDAC10 activity. Specifically, we indicated that HDAC10 function may be associated with many signaling pathways critical for the survival and proliferation of malignant T cells, including PI3K-Akt, JAK-STAT, and MAPK signaling pathways. PI3K-Akt/mTOR signaling is known to be hyperactivated in CTCL, and studies have shown the therapeutic potential of PI3K/mTOR inhibition in CTCL (Bresin et al., 2020). The JAK-STAT pathway is also known to be over-activated in various cancers, including CTCL, and contributes to malignant cell proliferation and survival (Brooks and Putoczki, 2020). Notably, the activation of STAT3 and STAT5 was observed in both early and late stages of CTCL, and upregulation of STAT5 at the beginning of the disease is correlated with increased expression of micro-RNA miR-155, that targets STAT4, which is important for proper T-helper 1 (Th1) differentiation, and this disruption is considered to be one of the molecular mechanism contributing to CTCL development (Netchiporouk et al., 2014). Moreover, our analysis emphasized the downregulation of *ACV1RC* upon HDAC10 overexpression. *ACVR1C* (Activin Receptor type 1C) is a gene that encodes a receptor involved in the transforming growth factor-beta (TGF-β) signaling pathway. It is correlated mainly with solid malignancies, including breast cancer (Du et al., 2024). Interestingly, the Activin Signalling Pathway is known to function as both–a tumor-suppressor and a tumor-initiator in cancer (Qiu et al., 2021), although this phenomenon was initially described for *ACVR1B* in pancreatic ductal adenocarcinoma (PDAC). In our analysis we observed gene downregulation in patient samples and cell lines overexpressing HDAC10, while *ACVR1C* was upregulated after HDAC10 knockdown. This may suggest the correlation between *ACVR1C* expression and HDAC10 modulation in SS and highlights the possible tumor suppressor nature of the activin signaling pathway. However, further research is required to understand the exact mechanisms underlying the presented observations.

As HDAC10 is revealed to be altered in SS, but no research has been ever conducted in this field, we decided to further examine its possible role in disease development or progression. Based on functional assays conducted on model SS cell lines, we indicated that overexpression of HDAC10 alone did not significantly impact biological processes like proliferation, cell cycle progression, or apoptosis, and this observation may be attributed to the endogenous expression of HDAC10 in SS cell lines, suggesting that additionally introduced overexpression of this histone deacetylase does not alter the cell biology., However, upon exposure to the pro-apoptotic compound Camptothecin (CPT), HDAC10-overexpressing cells presented an inhibitory effect on apoptosis progression after treatment, suggesting that HDAC10 may confer a survival advantage under apoptotic stress. Apoptosis has been studied in SS before, indicating epigenetic and genetic regulations, suggesting regulation of the programmed cell death processes by SATB1 and SNF5, but wide-observed mechanism of apoptosis resistance is still unknown (Li et al., 2018; Wang et al., 2011). In this study, we decided to apply the BH3 profiling technique to underscore the potential for apoptosis alterations upon HDAC10 overexpression in the malignant cells. BH3 profile revealed increased expression of the BCL2-related cell death agonist (BAD) in HDAC10-overexpressing Hut78 cells, suggesting its reliance on BCL2/BCL-XL and/or BCL-W apoptosis protection. This finding prompted us to explore the sensitivity of these cells to BCL2 family inhibitors. Surprisingly, despite the increase in BAD expression, HDAC10-overexpressing cells did not exhibit increased sensitivity to the BCL2 inhibitor venetoclax or the BCL2/BCL-XL/BCL-W inhibitor navitoclax. This lack of sensitivity may be because the increase in BAD expression in our SS cell lines is not sufficient enough to increase the direct susceptibility to these inhibitors. To give a comprehensive analysis of this gene in SS, we performed also the HDAC10 knockdown in Sézary cells, which marked a reduction in cell growth, together with the induction of apoptosis and autophagy processes, which highlights the potential of HDAC10 as a regulator of cell survival and death in SS. The induction of apoptosis and autophagy upon HDAC10 knockdown underlines the dual role of this gene in modulating cell fate, suggesting that targeting HDAC10 may disrupt critical survival mechanisms in Sézary cells, which potentates as a future therapeutic target. HDAC10 was indicated to play a diverse role in regulating cancer cell survival by influencing autophagy and apoptosis, processes that are often interconnected in determining cell fate (Cheng et al., 2021). Autophagy, a cellular recycling mechanism, is critical for maintaining homeostasis under stress conditions, such as nutrient deprivation or therapeutic intervention (Gómez-Virgilio et al., 2022). In cancer cells, HDAC10 has been implicated in facilitating autophagic flux, potentially by modulating the fusion of autophagosomes with lysosomes (Hai et al., 2021). This enables tumor cells to adapt to harsh microenvironments and evade apoptosis. For instance, studies in neuroblastoma have demonstrated that HDAC10 loss disrupts autophagy and sensitizes cancer cells to chemotherapy, underscoring its role in promoting survival under therapeutic stress (Oehme et al., 2013). In cancer cells, HDAC10 regulates Hsp70 activity, playing a significant role in promoting cell survival by supporting essential protective mechanisms (Oehme et al., 2013; Hai et al., 2021). By modulating autophagy through Hsp70 deacetylation, HDAC10 may help cancer cells survive under adverse conditions, but it could also push cells beyond their adaptive capacity, tipping the balance toward cell death. This presents a unique therapeutic opportunity where targeting HDAC10 could potentially enhance cancer cell vulnerability by disrupting their survival mechanisms, particularly in cancers that rely on autophagy for survival (Oehme et al., 2013; Hai et al., 2021). Hsp70’s involvement in autophagy is particularly crucial for cancer cells, helping them manage the stresses arising from rapid growth, chemotherapy, and harsh microenvironments (Ferretti et al., 2024). This interaction highlights the HDAC10 role in cellular stress responses and its potential as a therapeutic target. HDAC10 may suppress apoptosis by regulating the expression of key pro- and anti-apoptotic genes, fostering a pro-survival environment. This dual modulation of autophagy and apoptosis may position HDAC10 as an important player in cellular survival mechanisms in malignancies. However, autophagy’s dual role as both a survival mechanism and a pathway to autophagic cell death presents a unique opportunity - by targeting HDAC10, it may be possible to disrupt protective autophagic mechanisms, increasing cellular stress and enhancing the efficacy of conventional treatments such as chemotherapy. Importantly, in our study HDAC10 knockdown has demonstrated pro-apoptotic effects in SS cells, indicating a potentially favorable therapeutic index. These findings underscore the importance of further exploration into HDAC10-targeted therapies, including their potential synergy with agents that modulate autophagy or apoptosis, to effectively exploit the weaknesses of cancer cells.

Furthermore, our study showed that selective inhibition of HDAC10 increases the sensitivity of Sézary cells to the pro-apoptotic compound Camptothecin (CPT), even though the treatment with HDAC10i alone represented the relatively low potential of CTCL treatment due to low cytotoxicity. This finding is particularly significant because it suggests that inhibition of HDAC10 may enhance the efficacy of already existing chemotherapeutic agents, providing a combinatorial therapeutic strategy to overcome resistance in CTCL. Synergy between HDACi and other compounds, such as BET inhibitors (BETi), JAK inhibitors (JAKi), and BCL2 inhibitors (BCL2i) have been already indicated and described in the literature (Cyrenne et al., 2017; Yumeen et al., 2020; Kim et al., 2018). Published studies have demonstrated significant synergistic interactions between venetoclax and HDAC inhibitors (vorinostat and romidepsin) in the treatment of CTCL *in vitro*, which were dose-dependent and showed an antagonistic trend at lower doses and synergy at higher doses. Vorinostat was indicated to increase the expression of the pro-apoptotic genes (BCL2L11 and BMF) and also increased the efficacy of venetoclax (Cyrenne et al., 2017). This synergy extends to BET inhibitors, which when combined with venetoclax or HDAC inhibitors were detected to cause a dose-dependent reduction in CTCL cell viability and increased caspase 3/7 activation, correlated with apoptosis promotion (Kim et al., 2018). Combined treatment with BET inhibitors and HDAC inhibitors also significantly suppressed the MYC and BCL2 expression, which suggests a thesis of cooperative epigenetic regulation during synergistic combinations of HDACi. Additionally, JAK inhibitors presented increased efficacy when used in combination with BCL2, proteasome, BET, or HDAC inhibitors by enhancing apoptosis pathways (Yumeen et al., 2020). All these findings highlight the potential of synergistic combining HDAC inhibitors as a promising strategy for developing new therapies for CTCL, and the increased sensitivity to CPT following HDAC10 inhibition indicates that HDAC10 may play a protective role in Sézary cells. It would be interesting to further study how agents targeting BET, JAK, and BCL2 pathways work in combination with a specific HDAC10 inhibitor. Each of these classes of drugs addresses distinct yet overlapping mechanisms critical to CTCL pathogenesis, including transcriptional dysregulation, immune signaling, and apoptosis resistance so it is likely that they will work better together. And since HDAC10i is not cytotoxic alone, contrary to known pan-HDACi, it could enable dose optimization to maximize efficacy while minimizing toxicity. It is worth emphasizing that patients' response to such treatment may depend on the level of HDAC10 expression, similar to what has previously been confirmed for Bcl2 and JAK, where higher sensitivity to targeted drugs, Venetoclax and Ruxolitinib, respectively, was detected in patient samples with higher expression of those genes (Cyrenne et al., 2017; Yumeen et al., 2020). However, the response to the specific HDAC10i in targeted therapies may depend not only on the level of HDCA10 expression itself in a given patient but also on HDAC10 activity, and those may be influenced by specific genetic mutations observed in CTCL patients, particularly those affecting pathways that HDAC10 regulates or interacts with. Mutations in the JAK-STAT pathway could modify HDAC10’s functional role by altering the epigenetic landscape and transcriptional activity in CTCL cells (Choi et al., 2015). Similarly, mutations in epigenetic regulators like TET2 or DNMT3A could impact chromatin accessibility, potentially enhancing or suppressing HDAC10’s deacetylase activity (Iżykowska, 2021). Additionally, mutations in apoptosis-related genes such as FAS or TP53 might indirectly influence HDAC10’s role in regulating cell survival and death pathways, affecting its therapeutic potential (Chang et al., 2018). Understanding how these mutations intersect with HDAC10 activity could guide the development of more targeted and effective combination therapies.

The cytoplasmic localization of HDAC10, its involvement in cancer-related pathways, and its effects on cell growth, apoptosis, and autophagy make it a promising candidate for targeted therapy in SS. Future research should focus on developing selective HDAC10 inhibitors and exploring their synergistic effects with existing chemotherapeutic agents to enhance treatment efficacy and overcome resistance in CTCL.

## Data Availability

The datasets presented in this study can be found in online repositories. The names of the repository/repositories and accession number(s) can be found in the article/Supplementary Material.
