# Supplementary material for: HDAC10 and its implications in Sézary syndrome pathogenesis
Source: Front Cell Dev Biol. 2025 Jan 31;13:1480192. doi: 10.3389/fcell.2025.1480192 (PMC11825767; doi:10.3389/fcell.2025.1480192)
Supplement: Supplementary file 1 [file DataSheet1.pdf]

Supplementary Material

Supplementary Figures:

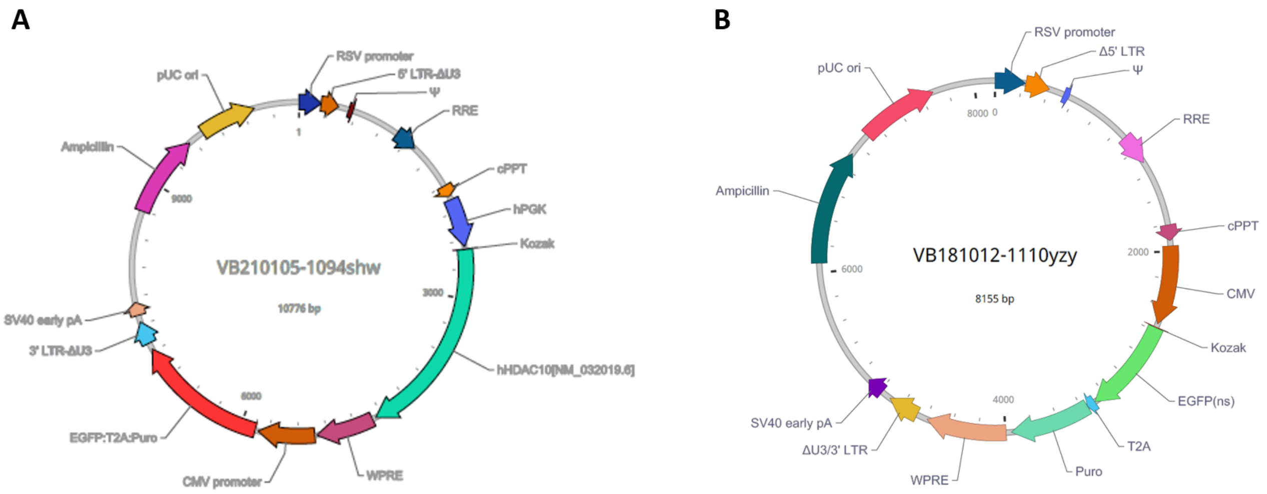

Supplementary Figure 1. Vector maps. (A) pLV[Exp]-EGFP:T2A:Puro-hPGK>hHDAC10[NM_032019.6]/3xFLAG (B) pLV[Exp]-CMV>EGFP(ns):T2A:Puro (Vector Builder)

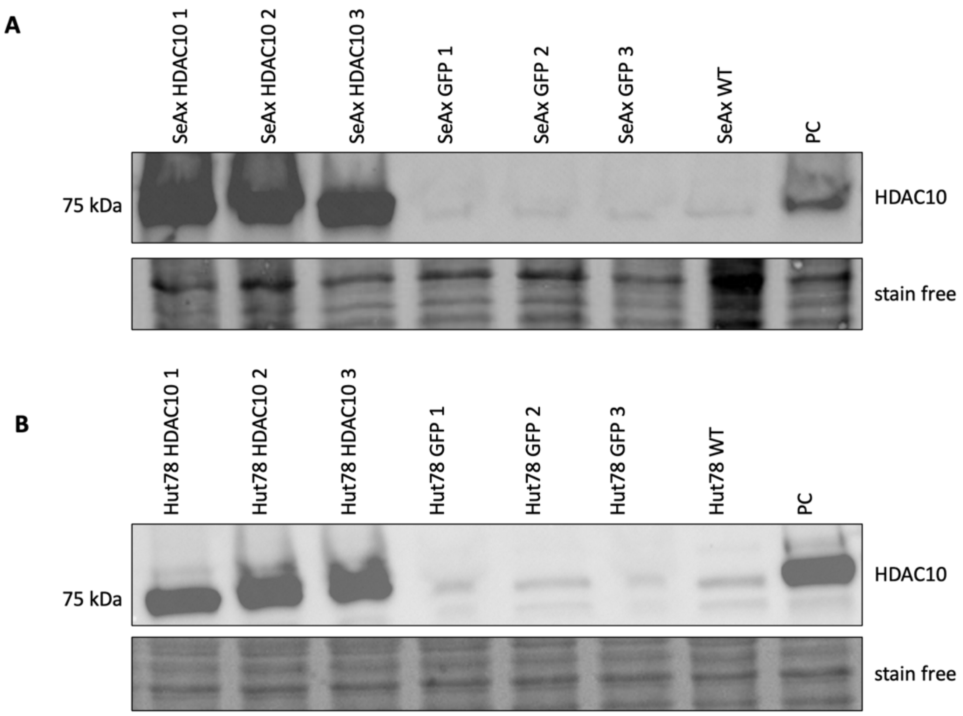

Supplementary Figure 2. Western blot analysis of HDAC10 expression in (A) SeAx and (B) Hut78 cell lines; SeAx HDAC10 1-3, Hut78 HDAC10 1-3: cell lines after introducing HDAC10 overexpression with lentiviral technology system with hPGK promoter; SeAx GFP 1-3, Hut78 GFP 1-3: control cell lines transduced with empty vector; PC – positive control (SeAx with HDAC10 overexpression). Predicted HDAC10 protein size– 72 kDa.

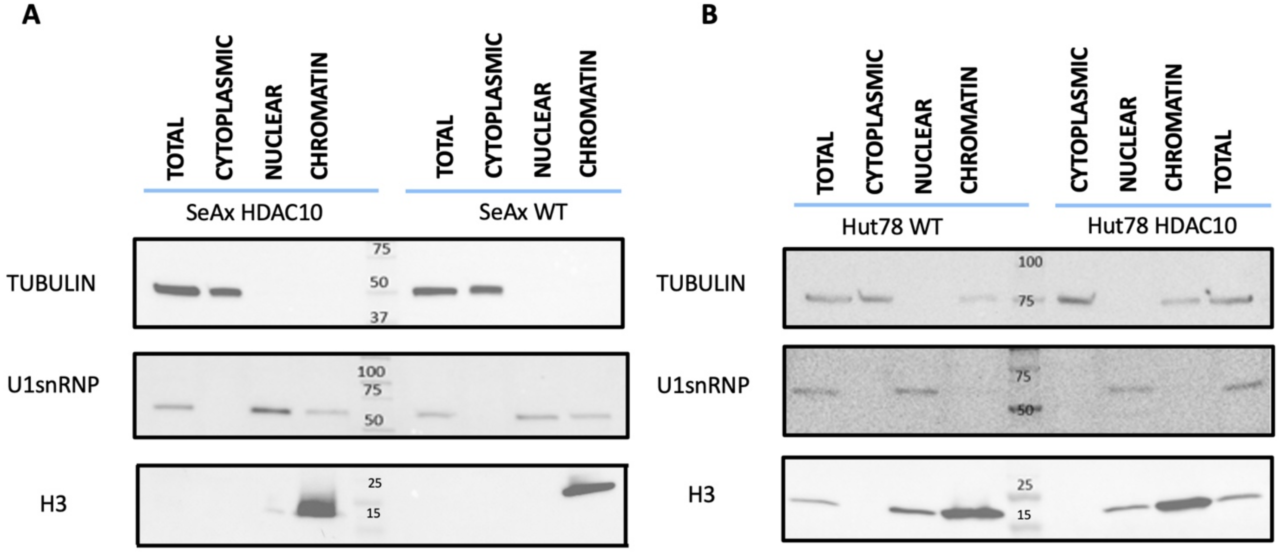

Supplementary Figure 3. Western blot analysis of cellular fractionation (A) Cytoplasmatic fraction – anti-βTubulin antibody (B) Nuclear fraction – anti-U1snRNP antibody (C) Chromatin fraction – anti-H3 antibody. Predicted protein size: βTubulin - 50 kDa, U1snRNP – 70 kDa, H3 – 17 kDa.

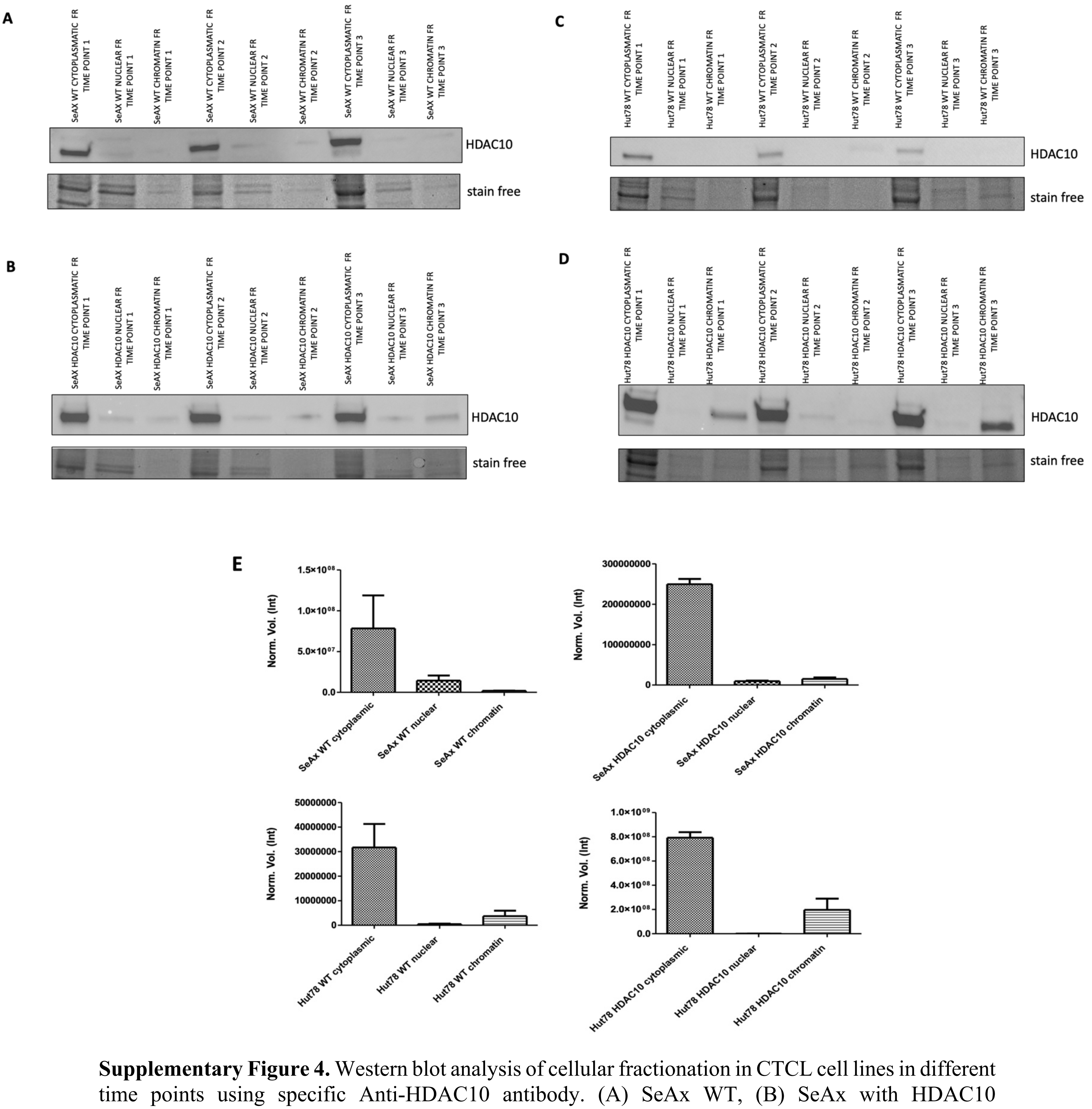

Supplementary Figure 4. Western blot analysis of cellular fractionation in CTCL cell lines in different time points using specific Anti-HDAC10 antibody. (A) SeAx WT, (B) SeAx with HDAC10 overexpression, (C) Hut78 WT, (D) Hut78 with HDAC10 overexpression. (E) Quantitative analysis of HDAC10 localization in SeAx WT, SeAx with HDAC10 overexpression, Hut78 WT and Hut78 with HDAC10 overexpression cell lines. The mean values ± SEM of three time points protein extraction are shown. Time point 1 – passage 4th. Time point 2 – passage 8th. Time point 3 – passage 12th.

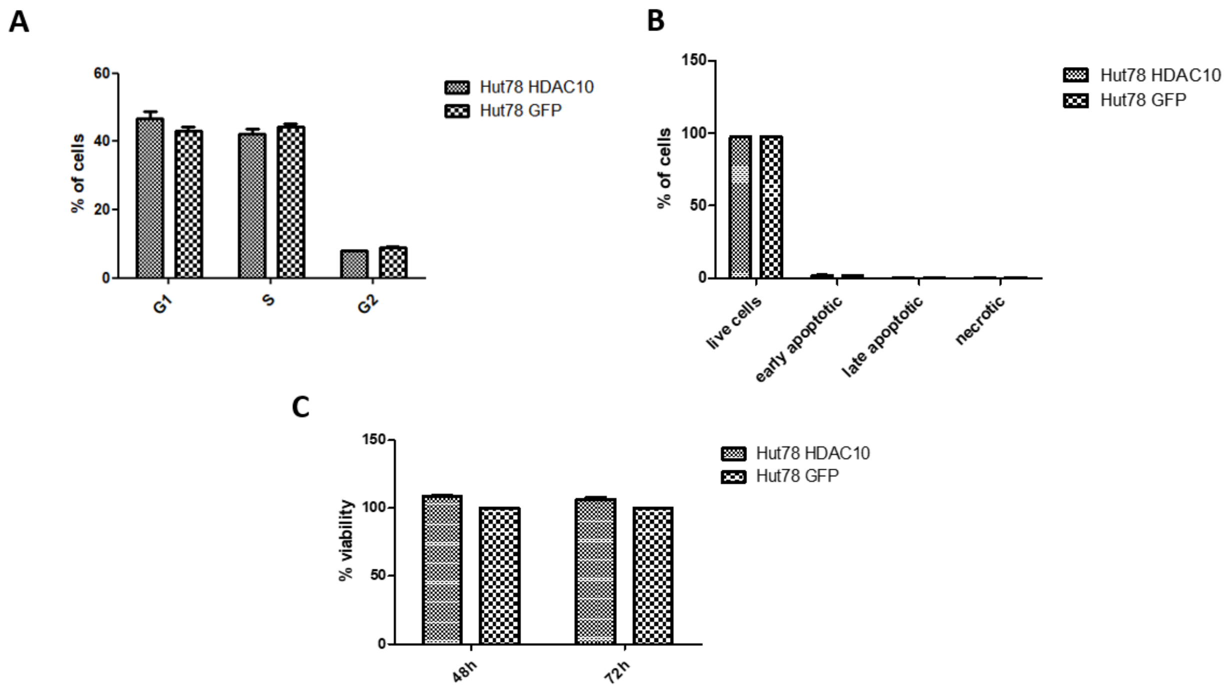

Supplementary Figure 5. Effect of HDAC10 overexpression on cell biology in Hut78 cell line. (A) Cell cycle analysis in Hut78 cells with HDAC10 overexpression and control using flow cytometry and BrdU/7AAD staining. The mean values ± SEM of 3 independent experiments are shown. (B) Apoptosis analysis in Hut78 cells with HDAC10 overexpression and control. The percentages of apoptotic, live, and necrotic cells were determined using flow cytometry and Annexin V/7AAD staining. The mean values ± SEM of 3 independent experiments are shown. (C) Cell viability upon HDAC10 overexpression in Hut78 cells. The mean values ± SEM of 3 independent experiments are shown.

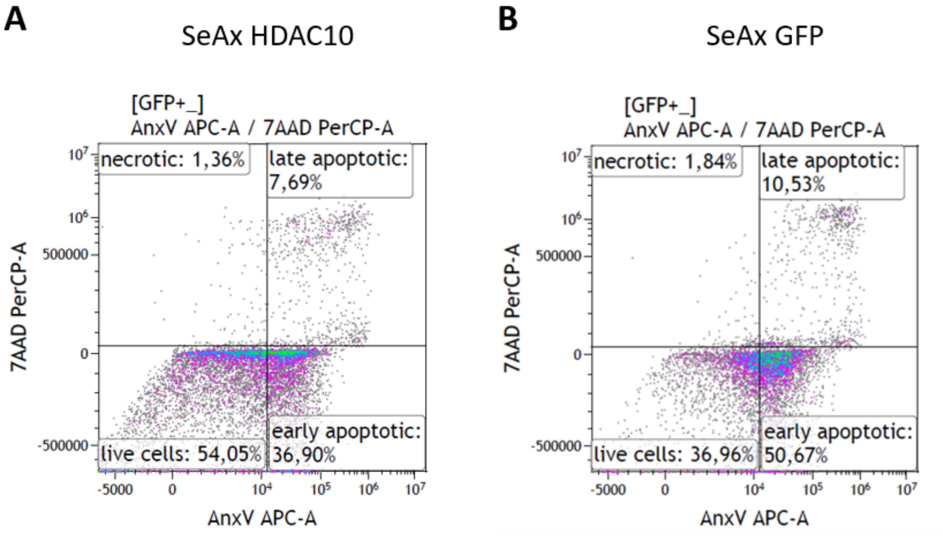

Supplementary Figure 6. Representative plots of apoptosis analysis using flow cytometry in (A) SeAx cell line with HDAC10 overexpression and (B) control.

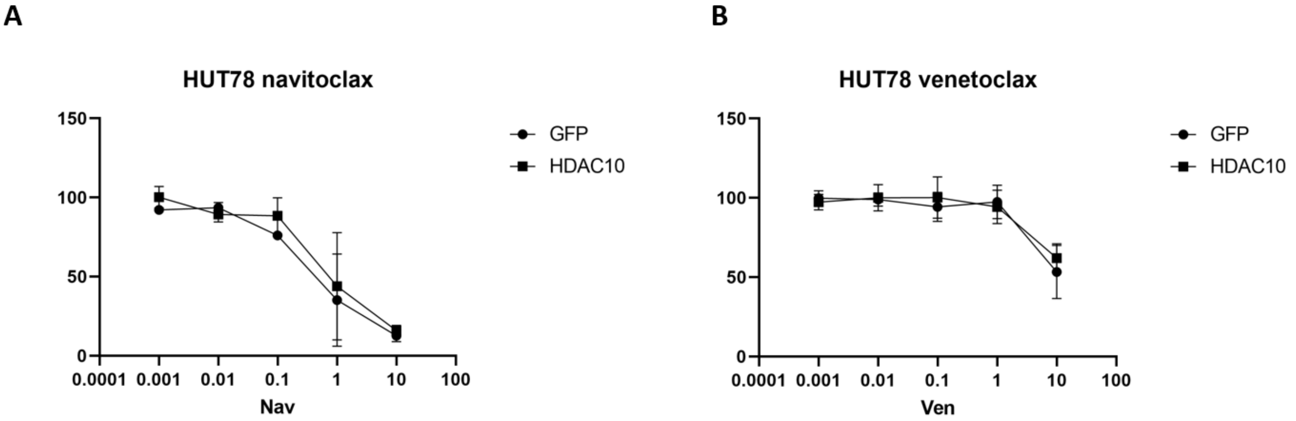

	GFP	HDAC10
IC50	0.4708	0.8361

	GFP	HDAC10
IC50	12.04	16.37

Supplementary Figure 7. BH3 mimetics sensitivity for Hut78 cell lines with HDAC10 overexpression for venetoclax (BCL-2i), navitoclax (BCL-2/XL/Wi). Sensitivity is measured by metabolic activity and normalized to untreated cells, IC_{50} values are given in µM if possible to calculate.

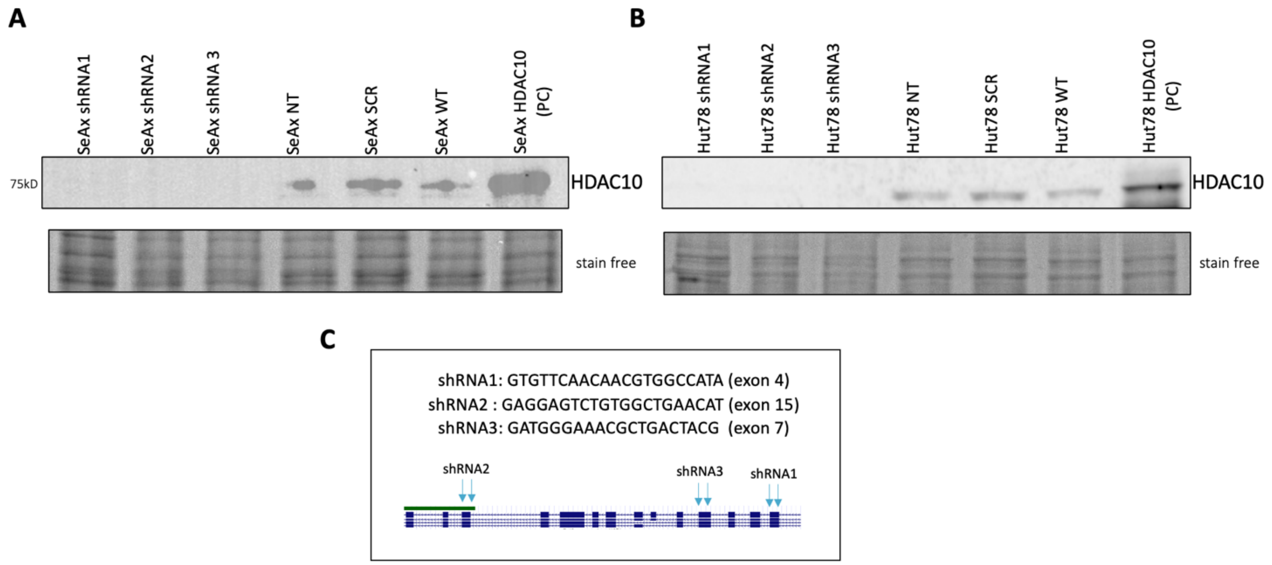

Supplementary Figure 8. HDAC10 silencing. Western Blot analysis of (A) SeAx and (B) Hut78 cell line after knockdown using designed HDAC10-targeting shRNAs. Predicted HDAC10 protein size– 72 kDa. (C) Visualisation of designed shRNA1, shRNA2 and shRNA3 and exons they are targeting and schematic illustration of the binding site for all shRNAs in the HDAC10 gene. NT - non targeting; SCR – scrambled; WT- wild type; PC – positive control

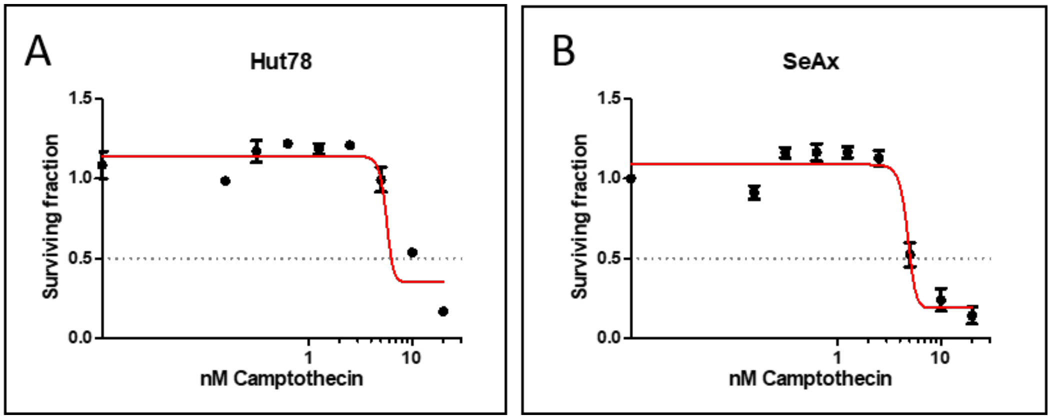

Supplementary Figure 9. IC50 values calculated for Camptothecin in CTCL cell lines, Hut78 (IC50=5.63) (A) and SeAx (IC50=4.77) (B); experiments performed in two biological and three technical replicates

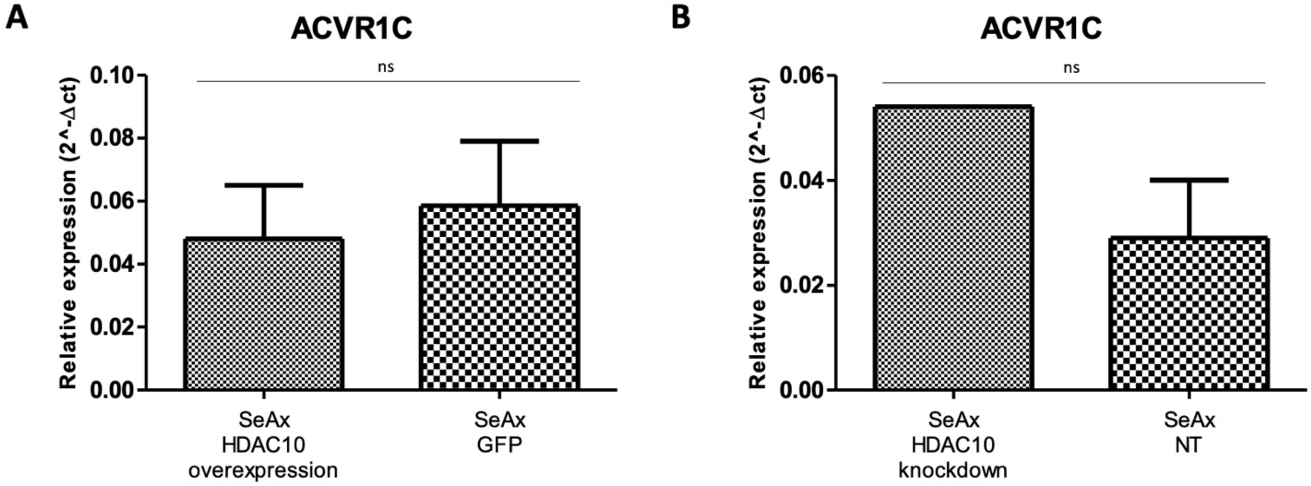

Supplementary Figure 10. qRT-PCR validation of ACVR1C gene expression in SeAx cell lines with HDAC10 overexpression (A), and HDAC10 knock-down (B) and corresponding controls (GFP- empty vector and NT - non-targeting).

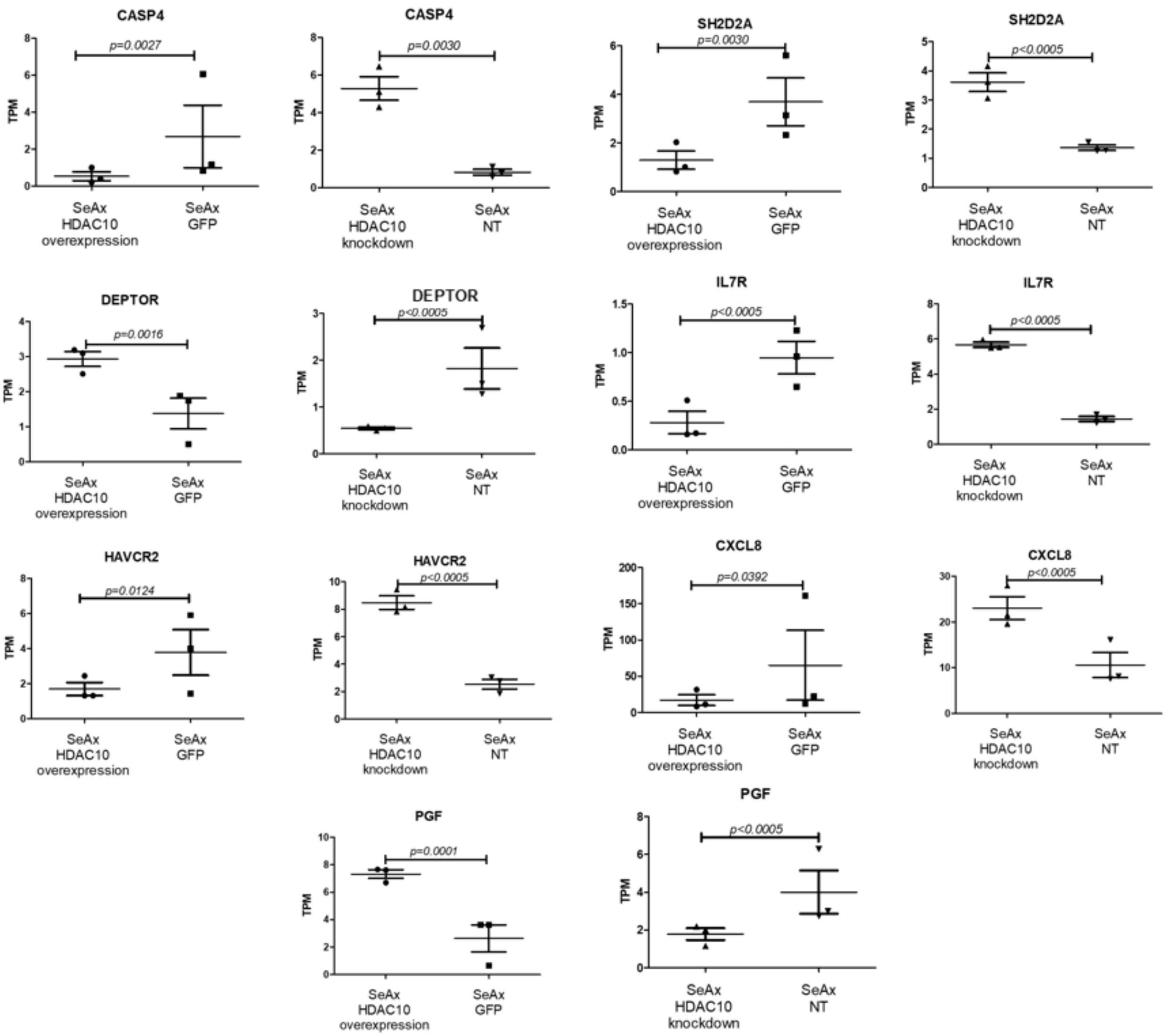

Supplementary Figure 11. Expression of selected genes in SeAx cell line with HDAC10 knockdown and HDAC10 overexpression, and corresponding controls; RNAseq analysis; raw.p<0.05; TPM (Transcripts Per Kilobase Million)

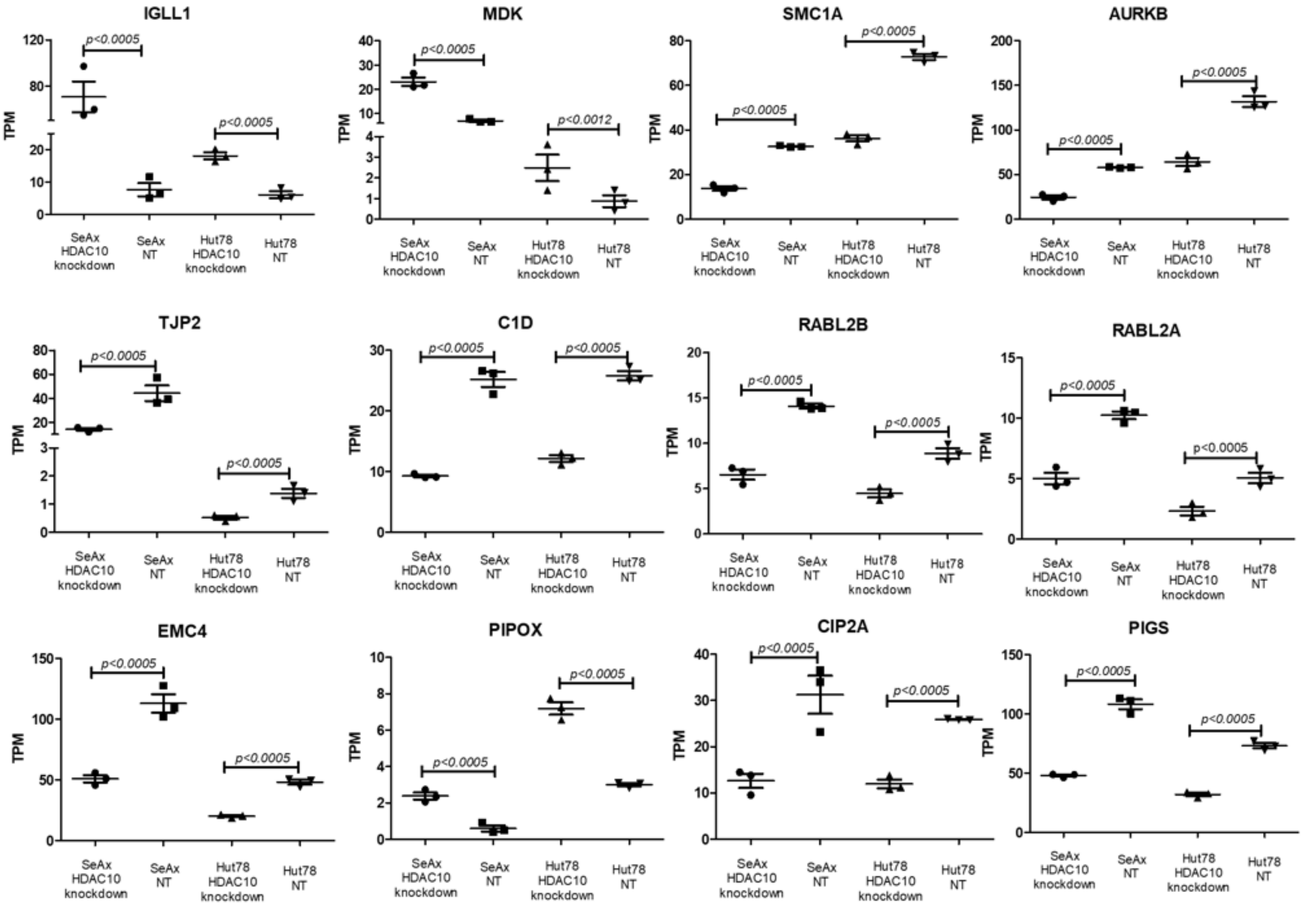

Supplementary Figure 12. Expression of selected genes in SeAx and Hut78 cell lines with HDAC10 knockdown, and corresponding controls; RNAseq analysis; raw.p<0.05; TPM (Transcripts Per Kilobase Million)

Supplementary Tables:

Supplementary Table 1. Clinical data

Patient	Diagnosis	Sex	Age	WBC count/nl	Lymphocytes count/nl [%]	atypical Sézary cells [%]	CD4/ CD8 ratio	CD4/ CD3	Therapy prior to sample collection
SS1	Sézary syndrome	F	74	28,15 x10^9/l	20,97 x10^9/l<br>74,5%	72,3	232,2:1	73,0%	pegIFN + bexaroten
SS2	Sézary syndrome	F	64	22,32 10^3/ul	16,24 10^3/ul	58	23:1	0,96:1	none
MF1	MF III B0	M	62	10,8 x10^9/l	1,46 x10^9/l<br>13,5%	0,7	2,3:1	9,0%	none
MF2	MF III B1	M	76	4,96 x10^9/l	1,06 x10^9/l<br>21,4%	13,8	5,8:1	17,3%	bexaroten

Supplementary Table 2. Genes with opposite expression patterns between the HDAC10 overexpression and knock-down in SeAx cell line; RNAseq DEG analysis, |fc|≥2 & raw.p<0.05

	SeAx HDAC10 overexpression/control.fc	SeAx HDAC10 knockdown/control.fc
MYRF	**-2,018537**	**2,914811**
CASP4	**-4,241892**	**6,048155**
GALNT3	**-2,424121**	**2,357129**
IL7R	**-3,134632**	**3,907393**
CXCL8	**-3,381320**	**2,195842**
IRAG2	**-3,271348**	**2,192924**
GPR65	**-4,538219**	**2,150531**
SH2D2A	**-2,544797**	**2,588618**
PI15	**-2,747446**	**2,199775**
HAVCR2	**-2,047063**	**3,351521**
ANTXR2	**-4,361272**	**3,154118**
ACVR1C	**-2,329375**	**2,464318**
BTNL9	**-3,059980**	**2,177575**
CFAP47	**-4,148369**	**2,258539**
C2orf66	**-2,182529**	**2,354716**
YWHAEP1	**-3,259829**	**3,265124**
MEOX1	**3,193700**	**-4,519136**
PGF	**2,987278**	**-2,218672**
PRG2	**4,957440**	**-2,588091**
SV2B	**2,134359**	**-2,965022**
MESP1	**4,022258**	**-2,382047**
KCNK13	**2,835841**	**-2,348252**
DEPTOR	**2,270732**	**-3,266699**
DOCK5	**3,853946**	**-3,585854**
RGS8	**6,498542**	**-5,890832**
CMTM8	**2,955734**	**-2,605556**
FER1L6-AS2	**2,911020**	**-3,380034**
C1QL3	**3,343918**	**-2,027805**
C1DP1	**4,247946**	**-6,272391**

Supplementary Table 3. Genes with the same expression patterns between the HDAC10 knock-down in SeAx cell line and Hut78 cell line; RNAseq DEG analysis, |fc|≥2 & raw.p<0.05

	SeAx HDAC10 knockdown/control.fc	Hut78 HDAC10 knockdown/control.fc
C3	**-5,092690**	**-2,711460**
GPC1	**2,065513**	**2,851828**
IGLL1	**9,023858**	**2,942724**
ITGB3	**-2,980285**	**-2,285276**
MDK	**3,251183**	**2,462924**
PLXNB3	**3,032805**	**3,344994**
RN7SL4P	**-2,946449**	**-2,071933**
TEAD3	**2,124641**	**3,479744**
THBS1	**-2,902804**	**-3,642556**
ZRSR2P1	**5,685698**	**4,257559**
SMC1A	**-2,405392**	**-2,036780**
AURKB	**-2,350604**	**-2,156097**
TJP2	**-3,084858**	**-2,460017**
C1D	**-2,716639**	**-2,151198**
SLC12A7	**2,260877**	**2,953624**
TSPAN9	**2,602889**	**3,354994**
RABL2B	**-2,197218**	**-2,039053**
RABL2A	**-2,024647**	**-2,128134**
EMC4	**-2,227096**	**-2,397505**
PIPOX	**3,891685**	**2,413411**
PCDHB14	**2,777964**	**4,066911**
PDZD4	**2,921937**	**2,053350**
CIP2A	**-2,312847**	**-2,039988**
HES4	**2,661800**	**3,500447**
DOCK5	**-3,585854**	**-3,086698**
ZMAT1	**2,775876**	**2,154040**
SLC35B4	**-2,087472**	**-2,019110**
RGS8	**-5,890832**	**-4,296900**
PIGS	**-2,264340**	**-2,311646**
TRIM16L	**-2,171866**	**-2,096724**
RN7SL2	**-2,687355**	**-2,707486**
RPL7AP39	**2,067700**	**2,033228**

Supplementary Table 4. Common deregulated KEGG pathways for selected groups; RNAseq analysis, $p < 0.05$

SeAx HDAC10 overexpression and SeAx HDAC10 knockdown	SeAx HDAC10 knockdown and Hut78 HDAC10 knockdown
Cell adhesion molecules	ECM-receptor interaction
Systemic lupus erythematosus	**PI3K-Akt signaling pathway**
Cytokine-cytokine receptor interaction	Focal adhesion
**Hematopoietic cell lineage**	Protein digestion and absorption
Neutrophil extracellular trap formation	Toxoplasmosis
Influenza A	Malaria
**Pathways in cancer**	Legionellosis
Rheumatoid arthritis	Human papillomavirus infection
Asthma	Regulation of actin cytoskeleton
Axon guidance	Rap1 signaling pathway
Ras signaling pathway	**Pathways in cancer**
Alcoholism	Phagosome
Pathogenic Escherichia coli infection	Efferocytosis
Viral carcinogenesis	**MAPK signaling pathway**
Pertussis	Neutrophil extracellular trap formation
Complement and coagulation cascades	Chemokine signaling pathway
Shigellosis	Proteoglycans in cancer
Staphylococcus aureus infection	
Viral protein interaction with cytokine and cytokine receptor	
Rap1 signaling pathway	
C-type lectin receptor signaling pathway	
Necroptosis	
**JAK-STAT signaling pathway**	
**MAPK signaling pathway**	
**NOD-like receptor signaling pathway**	
**Transcriptional misregulation in cancer**	
Intestinal immune network for IgA production	
**PI3K-Akt signaling pathway**	
Malaria	
Ether lipid metabolism	
Fc gamma R-mediated phagocytosis	
Choline metabolism in cancer	
Glycerophospholipid metabolism	
Protein digestion and absorption	
T cell receptor signaling pathway	
Coronavirus disease - COVID-19	
Toxoplasmosis	
Salmonella infection	
Inflammatory bowel disease	
Platelet activation	
Adherens junction	
Kaposi sarcoma-associated herpesvirus infection	
FoxO signaling pathway	
Linoleic acid metabolism	
EGFR tyrosine kinase inhibitor resistance	

Neuroactive ligand-receptor interaction<br>Phospholipase D signaling pathway<br>Human T-cell leukemia virus 1 infection<br>Primary immunodeficiency<br>Th1 and Th2 cell differentiation<br>Hepatitis B<br>AGE-RAGE signaling pathway in diabetic complications<br>Amoebiasis<br>Chagas disease<br>Human papillomavirus infection	

Supplementary Table 5. Deregulated genes in MAPK, JAK-STAT and PI3K-Akt signaling pathways in the SeAx cell line with HDAC10 overexpression and knockdown; RNAseq DEG analysis, $|fc| \geq 2$ & raw.p<0.05

SeAx HDAC10 overexpression		SeAx HDAC10 knockdown	
Genes	control.fc	Genes	control.fc
**MAPK signaling pathway**			
RASGRP2	2,374613	DUSP2	-2,402099
CSF1	2,059377	DUSP8	2,631747
DUSP6	-2,578434	FGFR3	2,454421
PGF	2,987278	FOS	-5,476761
PLA2G4A	-2,177545	FGF20	-2,776958
TGFB3	2,740054	IL1R1	2,099974
TNFRSF1A	-4,058045	PAK1	2,769554
FGF17	2,720293	PGF	-2,218672
CACNA1H	2,279759	PLA2G4A	-3,683248
		TGFBR2	2,839845
		CACNB1	2,198911
		PLA2G4C	2,295047
		CACNA1H	2,016517
**JAK-STAT signaling pathway**			
IL19	-3,651195	CLCF1	4,614241
IL7R	-3,134632	IL5	-2,717637
IL9	-15,918240	IL6	-2,032311
IL10	-5,106895	IL7R	3,907393
OSM	-2,400829	IL10	-3,541407
PRL	2,633390	IL12A	-2,581688
PTPN6	-2,403093	IL15	2,078308
		LIFR	2,262076
		IL26	3,763388
**PI3K-Akt signaling pathway**			
COL4A6	3,114376	BCL2L11	2,039555
CSF1	2,059377	COL4A6	2,524277
GNB3	2,258298	COL6A1	2,343139
IL7R	-3,134632	FGFR3	2,454421
OSM	-2,400829	COL6A5	2,298985
PGF	2,987278	FGF20	-2,776958
PRL	2,633390	TNC	3,570852
TNXB	2,802099	IL6	-2,032311
FGF17	2,720293	IL7R	3,907393
		ITGA2B	2,178352
		ITGB3	-2,980285
		LAMB3	2,377539
		LAMC2	3,104024
		MYB	-2,074491
		PGF	-2,218672
		PPP2R5B	2,075757
		THBS1	-2,902804
		LPAR2	2,419104

Whole blots (membranes)

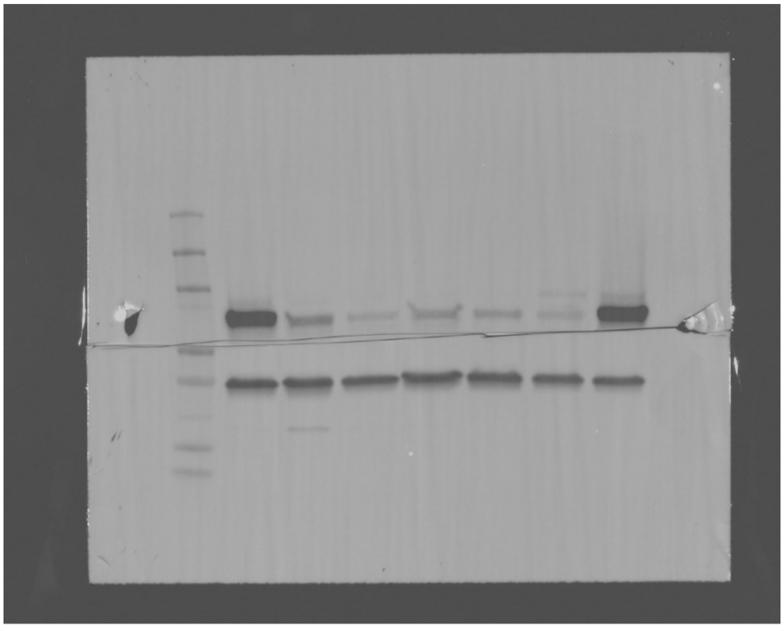

Figure 1.

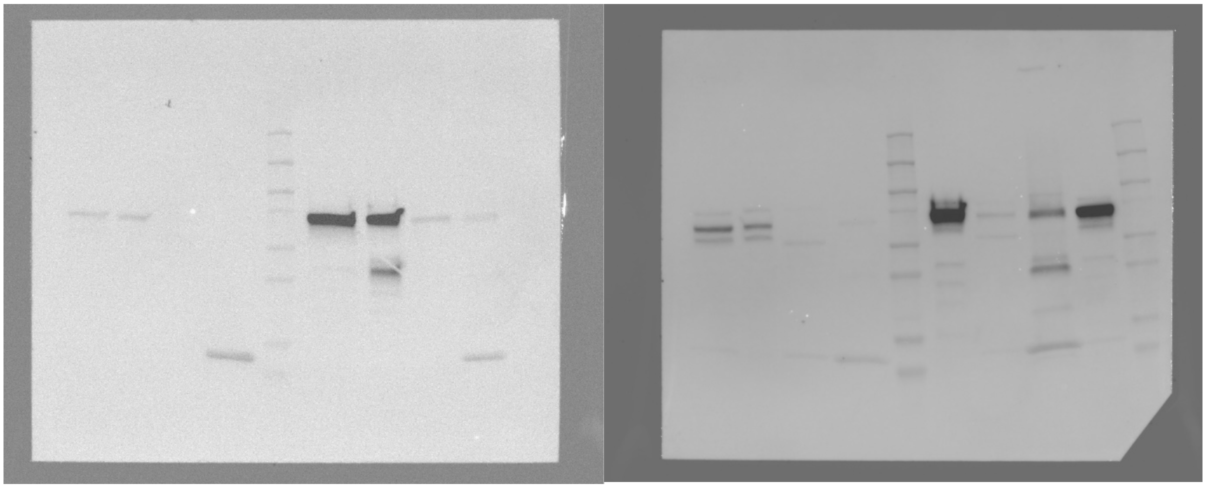

Figure 2.

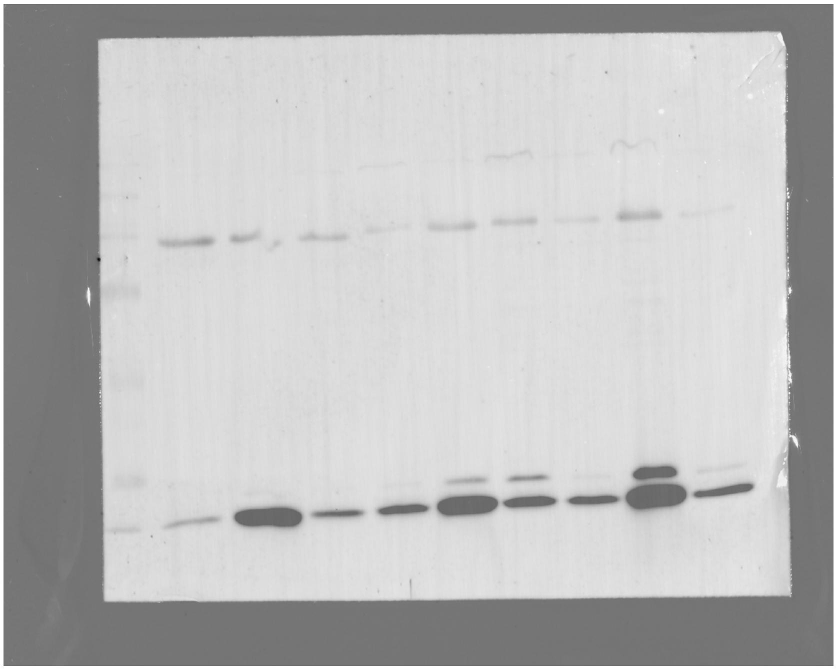

Figure 6.

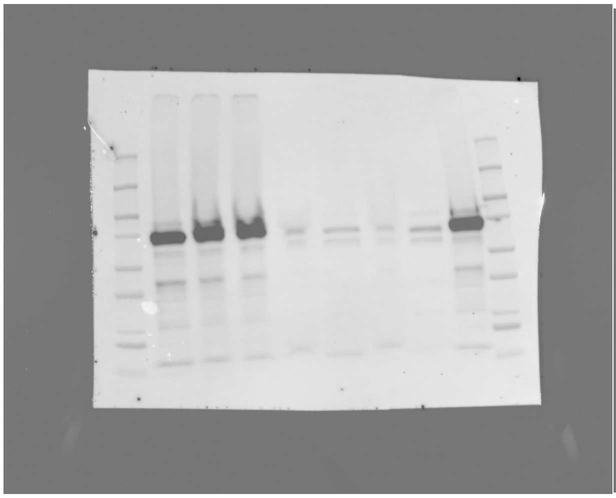
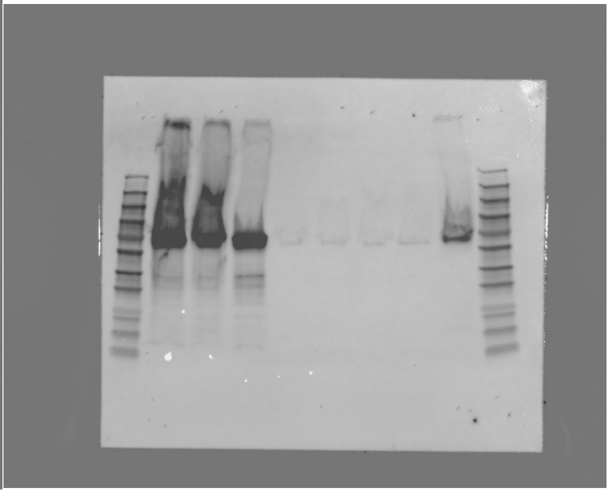

Supplementary Figure 2.

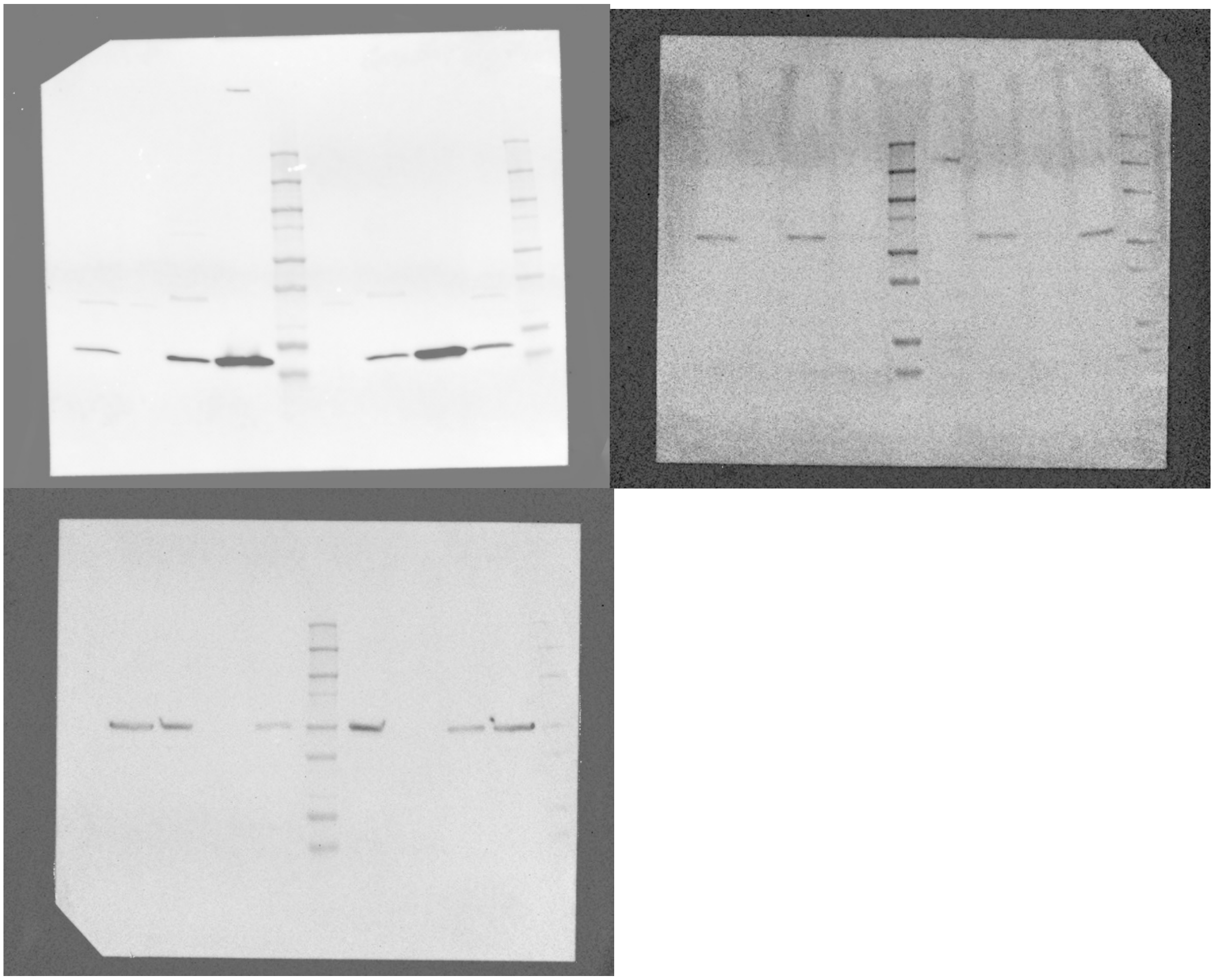

Supplementary Figure 3.

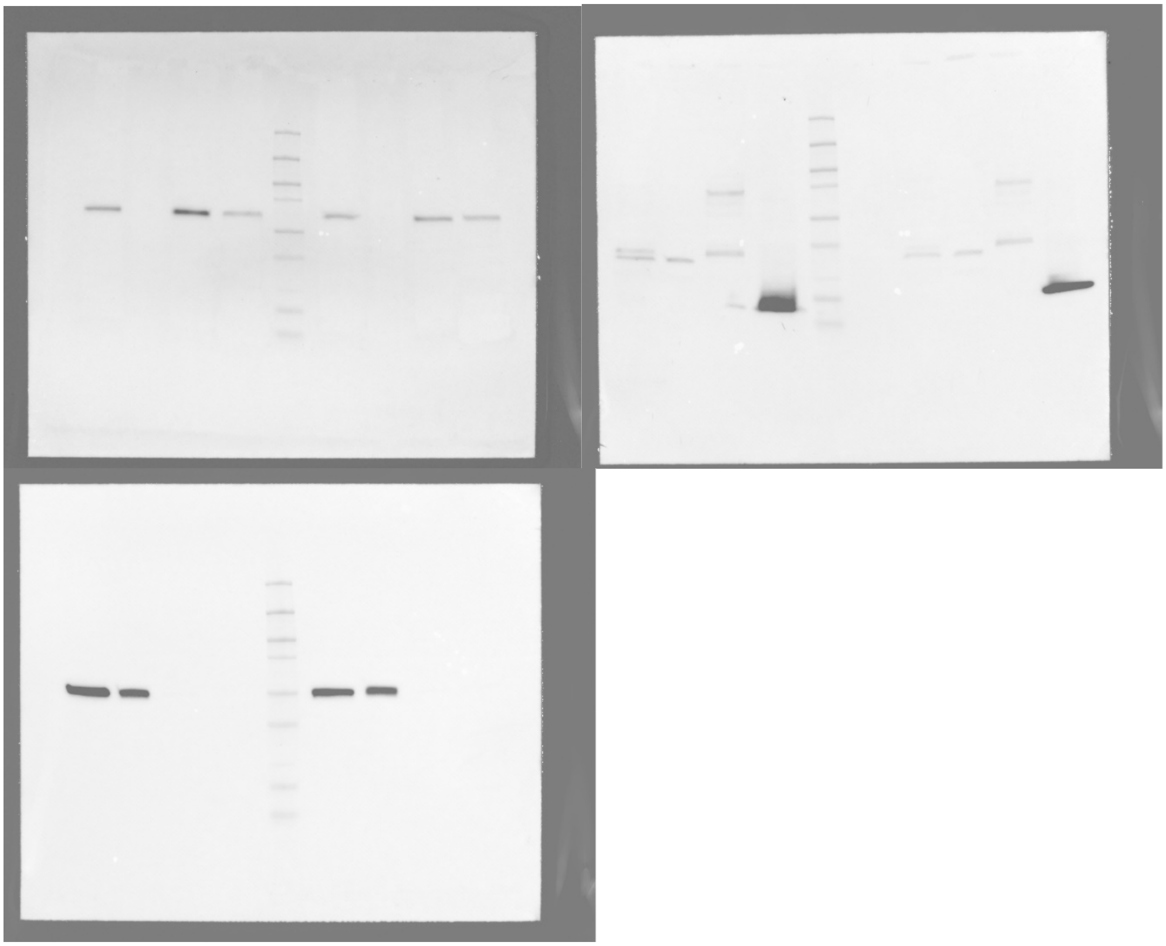

Supplementary Figure 3.

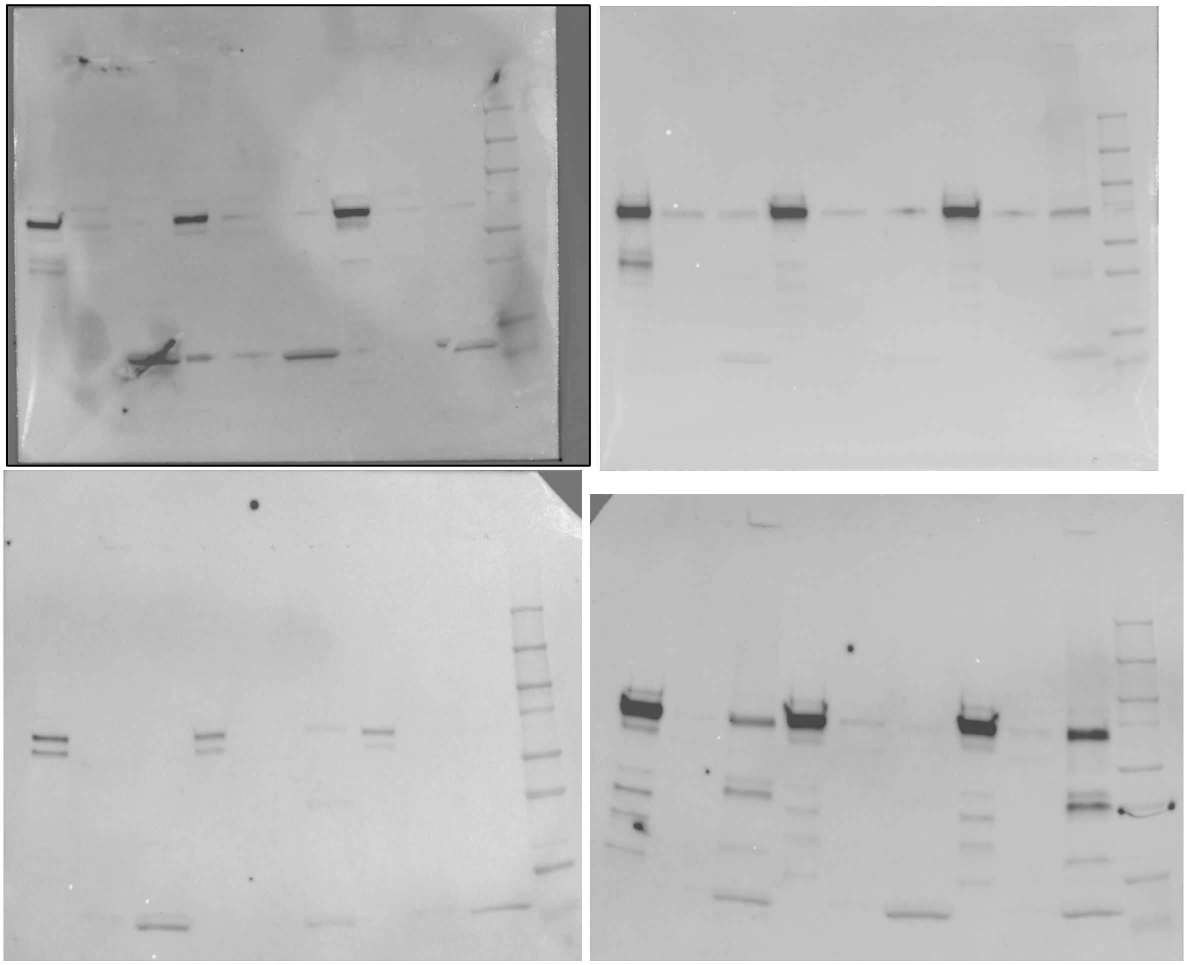

Supplementary Figure 4.

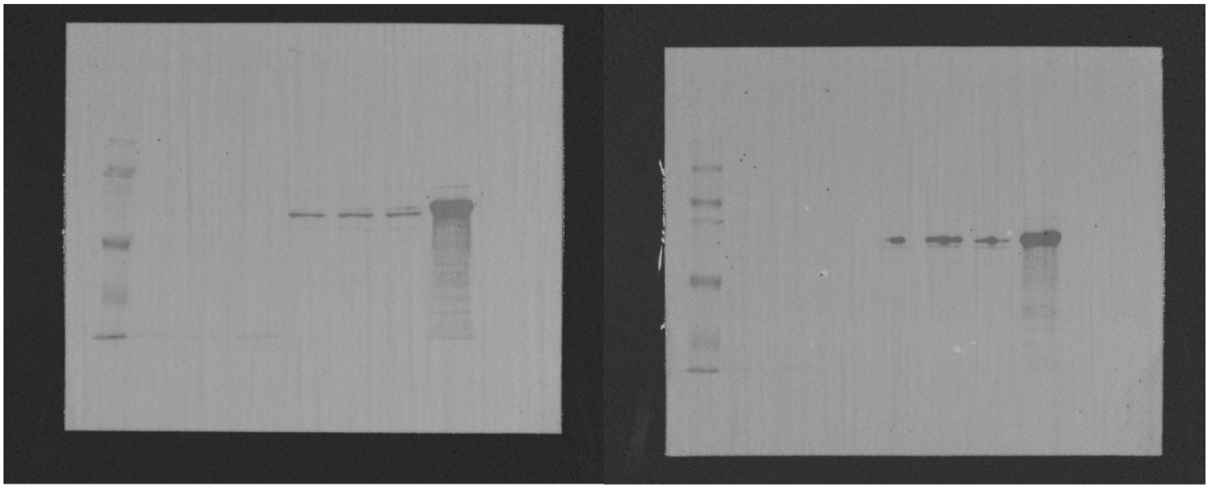

Supplementary Figure 8.
